# Preliminary results on the control of *Aedes spp.* in a remote Guatemalan community vulnerable to dengue, chikungunya and Zika virus: community participation and use of low-cost ecological ovillantas for mosquito control

**DOI:** 10.12688/f1000research.8461.3

**Published:** 2017-02-22

**Authors:** Gerard Ulibarri, Angel Betanzos, Mireya Betanzos, Juan Jacobo Rojas

**Affiliations:** 1 Department of Chemistry and Biochemistry, Laurentian University, Sudbury, Canada; 2 Instituto Nacional de Salud Publica, Morelos, Mexico; 3 Area de Salud Suroccidental-Sayaxche, Sayaxché, Guatemala

**Keywords:** Zika virus, mosquito-borne disease, virology, viruses, vector control, Latin America, community engagement, Dengue virus

## Abstract

**Objective:** To study the effectiveness of an integrated intervention of health worker training, a low-cost ecological mosquito ovitrap, and community engagement on *Aedes* spp. mosquito control over 10 months in 2015 in an urban remote community in Guatemala at risk of dengue, chikungunya and Zika virus transmission.

**Methods:** We implemented a three-component integrated intervention consisting of: web-based training of local health personnel in vector control, cluster-randomized assignment of an ecological modified ovitrap (ovillantas: ovi=egg, llanta=tire) or standard ovitraps to capture *Aedes spp.* mosquito eggs (no efforts have been taken to determine the exact *Aedes* species at this moment), and community engagement to promote participation of community members and health personnel in the understanding and maintenance of ovitraps for mosquito control. The intervention was implemented in local collaboration with Guatemala’s  Ministry of Health’s Vector Control Programme, and in international collaboration with the National Institute of Public Health in Mexico.

**Findings: **Eighty percent of the 25 local health personnel enrolled in the training programme received accreditation of their improved knowledge of vector control. When ovillantas were used in a cluster of ovitraps (several in proximity), significantly more eggs were trapped by  ecological ovillantas than standard ovitraps over the 10 month (42 week) study period (t=5.2577; *p*<0.05). Repetitive filtering and recycling of the attractant solution (or water) kept the ovillanta clean, free from algae growth. Among both community members and health workers, the levels of knowledge, interest, and participation in community mosquito control and trapping increased. Recommendations for enhancing and sustaining community mosquito control were identified.

**Conclusion:** Our three-component integrated intervention proved beneficial to this remote community at risk of mosquito-borne diseases such as dengue, chikungunya, and Zika. The combination of training of health workers, cluster use of low-cost ecological ovillanta to destroy the second generation of mosquitoes, and community engagement ensured the project met local needs and fostered collaboration and participation of the community, which can help improve sustainability. The ovillanta intervention and methodology may be modified to target other species such as *Culex*, should it be established that such mosquitoes carry Zika virus in addition to *Aedes*.

## Introduction

There is increasing concern about mosquito-borne disease, amplified by the recent Latin American outbreaks of Zika virus, which have raised new alarm about their rapid spread and illness in vulnerable populations ^1^. The fact that globalization increases vector migration has been demonstrated with the first finding of mosquitoes infected with the African West Nile virus in 2009 in the New York City region ^2^. The Americas have seen invasion of dengue virus, chikungunya virus, and most recently the Zika virus, causing dangerous outbreaks and subsequent morbidity and mortality that have proved difficult to control in a sustainable manner.

All of these viruses, plus the yellow fever virus, are transmitted mainly by mosquitoes of the *Aedes* genus (subgender: *Stegomyia*), more specifically the African species *Aedes aegypti*, one of the most aggressive vector mosquitoes capable of transmitting these illnesses, and to a lesser level the Asian tiger mosquito, *Aedes albopictus*, which prefers to bite during the day and is emerging as one of the most adaptable insects in the world ^3– 5^. The rapid rise of virus transmission on the American continent is due partly to the lack of historical immunological experience (have not been exposed to the viral infection) or known cross-immunization to the different species and viral genotypes ^6, 7^ of the people in the Americas. A second factor affecting the transmission is the stationary abundance of the vector *Ae aegypti* and other species susceptible to viral infection (e.g. *Culex spp?*). Furthermore, the traditional control method – pesticide – is both indiscriminate and thwarted by the multiple cases of pesticide resistance in *Aedes* mosquitoes recently reported ^8, 9^.

It is believed that *Ae aegypti* mosquitoes arrived to America with the Europeans during the colonization on the 16 ^th^ century ^10a^. It was Cuban Dr. Carlos Juan Finlay, working with Dr. Walter Reed, who identified the *Ae. Aegypti* as the culprit of the yellow fever outbreaks in Haiti and Panama in the 19 ^th^ century ^10b^, The rapid spread of several viri to most of the American continent began around the year 2000 ^10c^. Now a days dengue fever is active in more than 128 countries ^11^, chikungunya virus in 40 countries ^12^, and the Zika virus in currently 26 countries and rising ^13^. So far, we have not been able to stop it’s advance or vector potential. What solutions are available?

**Figure 1.**
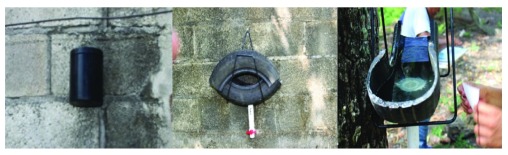
Recycling of a tire to reduce the *Aedes spp.* mosquito using directed oviposition. **a**) Classical ovitrap **b**) Covered ovillanta **b**) Inside an ovillanta showing the landing strip.

Several methods have been employed for detecting the presence of mosquitoes in the field, but none have proved perfect. Standard ovitraps to monitor *Aedes* mosquitoes are large 1-litre black buckets ( Figure 1) filled with plain water or attractant solutions based on natural plant infusions, along with a wooden strip or porous pellon paper, which the mosquito lands on and lays its eggs (called oviposition) ^14, 15^.

Human landing techniques have also been used, but less so now due to ethical concerns. The BG-Sentinel trap is a newer mosquito monitoring device, which has proved similar or better to the human-landing method for the detection of mosquitoes such as *Anopheles darlingi* ^16^.

Of these monitoring methods, only the standard ovitraps have thus far proved to effectively reduce the amount of adult mosquitoes when they are used for extended periods and the ovitraps are continually surveyed and maintained, a very expensive and time-consuming exercise ^17^.

To overcome these limitations, other ovitraps have appeared on the market – some force the larvae to a confined, flooded partition where they die because they cannot reach the surface and breathe. Others contain glue or larvicidal compounds, chemical or biological (e.g. *Bacillus thuringensis,* fish ‘ *Poecillia maylandi*’), to rapidly destroy the larvae. Each of these new traps has monitoring merits, but none have proved efficacious in the control and reduction of mosquitoes in field studies ^18^.

Typically, the water or attractant solution used in these ovitraps is removed and expelled into the ground each time the ovitrap is surveyed, and as a result any larvae or pupae are destroyed on the dry soil. The collection of the eggs “glued” to the landing strip allows for counting and thus monitoring. Two problems have been identified with this “dumping” of the solution. First, the need for clean replacement water is often problematic in remote areas without the basic infrastructure, and second, any eggs dislodged from the landing strip onto the dumped water or solution represent the possibility of continuous reproduction of mosquitoes. It is known that *Aedes* eggs can survive on the dry ground for several months ^19^, thus creating a compromised situation of perpetual reproduction.

To address these problems, we determined that filtration and recycling of the solution (either water or attractant) seemed to be an optimal alternative, and we set about developing a modified ovitrap. The most attracting solution to *Aedes* mosquitoes is the solution that has had larvae of the same species (conspecific larvae), making the recycling of the solution more attractive to female mosquitoes ^20^.

The development of our modified ovitrap at Laurentian University in Sudbury, Canada was initially geared to the reduction of the local West Nile virus vector, mainly the species *Culex pipiens* and *Culex restuans*. A 90% selective reduction of the adult mosquitoes of these species was achieved over three months around the site where the ovitraps were installed in 2008 (unpublished report, Ulibarri *et al*.). The attractant solution used in these modified ovitraps was based on the fermentation of natural plants spiked with homemade chemicals known to be oviposition-attractive to these species, which we also developed. The modified ovitrap is commercially available in Canada (Green Strike; http://green-strike.com/products/mosquito-preventer-2) together with the solutions that selectively attract the local *Culex* spp. (Cu-Lure™) or *Aedes* spp. mosquitoes (Ae-Lure™) (e.g *Aedes vexans/Ochlerotatus canadiensis*). The attractant has the advantage of being both non-toxic and environmentally sensitive.

The same principles driving the Canadian ovitrap were later used in a field study during 2013-2014 in Petatlán, Mexico against the dengue fever mosquitoes *Ae albopictus* and *Ae aegypti*. The *Aedes* oviposition was reduced by 71% on the site with the modified ovitraps, compared to oviposition in a control site (unpublished report, Ulibarri *et al*.).

Building on these previous studies, we undertook a field study in Sayaxche, Guatemala beginning in February 2015. We were determined to use an integrated approach that involved education of the community and health workers responsible for vector control about the mosquito cycle, sustainable strategies for keeping homes and gardens clean and unattractive to mosquitoes, the implementation and maintenance of mosquito traps, and collaboration between community and health sectors to collectively manage vector control and prevention of mosquito borne disease. Here we describe our project and its results.

When we embarked on the collaborative project, two challenges arose immediately. First, we discovered that local health workers required more extensive training in vector control than initially thought. This was promptly solved when the Instituto Nacional de Salud Publica (INSP) in Cuernavaca, Mexico offered to share a beta version of a web-based programme they created with the International Development Research Centre (IDRC) in Canada, to provide vector control and health information in Spanish (see Annex 1).

Second, we were unable to procure our original ovitraps from Canada, and we needed to search for an appropriate alternative. We decided to use recycled tires – partly because tires represent up to 29% of the breeding sites chosen by *Ae aegypti* mosquitoes, and it also gave us the opportunity to recycle some old tires that were littering the local environment ^21, 22^. Modifying the ovitrap with tires gave birth to the ovillanta, a piece of a tire fitted with a valve to help direct the attractant solution to a filter ( Figure 1).

## Methods

For this project, we designed the intervention to include three components in an integrated fashion: training health workers in vector control, low-cost ecological mosquito ovillanta, and community engagement. We sought to determine the effectiveness of this integrated intervention on *Aedes* spp. mosquito control over 10 months in 2015 in an urban remote community in Guatemala at risk of dengue, chikungunya and Zika virus transmission.

Our project was enabled by intersectoral collaboration between academic researchers, local health authorities from the Ministry of Health Vector Control Programme of Guatemala. international collaborators in Canada, Guatemala and Mexico, and community members. Our broad goal was to empower the community, supported by trained health personnel, to adopt the administration, care and maintenance of the health of the community.

***Ethics.*** This study was reviewed and authorized by the representatives of the Program on Vector-Borne Transmitted Diseases of the Ministry of Public Health and Social Assistance of Guatemala (Programa de Enfermedades Trasmitidas por Vectores del Ministerio de Salud Publica y Asistencia Social (ETV/MSPAS) de Guatemala). The MSPSA follows guidelines adopted from the Reglamento del Sub-Comité de ética e investigación ( www.paho.com) ^23^. We interviewed and invited each of the health workers to participate in the web-based training and encouraged them to voluntarily subscribe online to the program. We personally engaged people within the community, always accompanied by workers from the Health Unit of Sayaxche. It is customary in the region to first engage the representatives of the community by a verbal invitation to sit down and discuss the project. Once permission was obtained, we randomized the houses, and invited adults to voluntarily participate, assuring them that if there was anything that caused discomfort we would withdraw them immediately from the program. All invitations, focus groups, and interviews were verbally carried out in Spanish and recorded on a portable recorder, and transcribed soon after. When necessary, an interpreter was present for interviews with non-Spanish speaking community members. We, accompanied by representatives of the community leaders, explained the benefits of their participation and their responsibilities, allowing them to voluntarily join the study project. The protocol for the community engagement/social participation evaluations was also presented and approved by the ETV/MSPAS representatives.

***Study area.*** Sayaxche is a remote urban center of the southwestern part and health unit area of the Peten territory in Guatemala. It borders the states of Chiapas and Tabasco in Mexico. Sayaxche’s Q’eqchi Maya origin means the ‘Ceiba’s wye or fork.’ The surface is 3752 km ^2^ and occupies 10.9% of Peten’s territory. It is 250 m above sea level, with a warm, varied and humid tropical climate, with a rainy season (June to November) and a dry season (December to May). The monthly average temperature varies between 23°C (December/January) and 32°C (in the dry month of May).

***Intervention.*** Our three-component integrated intervention consisted of: a) web-based training of local health personnel in vector control, b) cluster-randomised assignment of ecological ovillantas or standard ovitraps to capture *Ae aegypti* mosquitoes, and c) community engagement to promote participation of community members and health personnel in the understanding and maintenance of ovitraps for mosquito control.

**a) Web-based training of local health personnel in vector control ( Annex 2).** As we embarked on our project we consulted with the local health authorities, including the manager of the Vector Control Programme in Sayaxche, to determine the needs of the personnel. These included the need for improved technical skills, liaising with community including cultural sensitivity, and promoting and implementing programmes, as well as a strong need to improve the efficacy of the preventive measures of vector transmission in the region. As mentioned earlier, the health workers had higher educational needs than we had initially expected.

To meet these needs, we collaborated with academic leaders at the INSP and with colleagues in the EcoHealth Leadership Initiative on Vector Borne Diseases for Latin America and the Caribbean, which is part of IDRC. We developed learning objectives, a web-based platform and a bibliography of resources to help strengthen the technical skills of the health personnel in the vigilance, prevention and control of vector-borne diseases using an ecosystem approach that aims for sustainability and is tailored to local community needs (see Annex 1 and Annex 2 for more details of the curricular planning). The web-based training modelled a dynamic process of teaching-learning, incorporating both theoretical and practical aspects of how to prevent and control vector-borne diseases. It utilized diverse learning strategies with specific content sessions, directed homework, and literature review. It took place over 5 months (April to September) in 2015, covering 40 hours in total, with 50% of practice and homework on a virtual platform.

We measured improved knowledge and skills of local health personnel in vector control by their successful accreditation following the web-based training programme.

**b) Control of vector mosquitoes using ovillantas.** We developed an ecological ovillanta (a modified ovitrap: ovi=egg, llanta=tire in Spanish) from recycled tires, and compared this device to standard ovitraps in terms of mosquito oviposition. We quantified egg collection from standard ovitraps on sites with ovillantas and compared it to standard ovitraps on sites without ovillantas.

We modified the standard ovitrap to create ovillantas, made out of a piece of recycled tire with a PVC flanged wash basin drain-type tube and a valve, for the capture of the vector mosquito’s eggs ( *Ae aegypti*). The attractant solution called *AE*-Lure (8mL) was placed on the ovillanta and two litres of clean well water were added. Each ovillanta contained two landing strips – one on each end of the apparatus. For the landing strips we used 15×10 cm pieces of pellon paper, but other porous material might be used.

We compared the ecological ovillantas with the standard ovitrap, which was a black bucket containing one litre of clean well water with one piece of the same pellon paper (but of a larger size, 30×10 cm) around the rim.

The urban core of Sayaxche contains 15 neighbourhoods, which comprised 14,454 inhabitants within 3,882 houses. We designed the random sampling based on our previous study in Petatlán, Mexico (unpublished report, Ulibarri *et al*.) and modified it to fit Sayaxche, applied as follows: 14 neighbourhoods (out of 15) were divided in half making two separate groups, the study group and the control group. Within each neighbourhood, 3 continuous blocks of houses were randomly chosen and one house at the center of each side of the block (north, east, south and west) was consulted to set up either 2 ovillantas and 1 standard ovitrap (study site) or 1 standard ovitrap per house (control site). If a chosen house declined to participate, the next house was invited. In the end each neighbourhood had 12 households participating. The total number of sites with ovillantas/ovitraps were 84 for the study group and 84 for the control group (see Table 1 for the names of the neighbourhoods).

**Table 1.** Description of neighbourhoods and control sites in Sayaxche, Peten, Guatemala.

Study sites (Ovillantas)			Type of ovitrap	Distribution		Total
Name	# Houses	Inhabitants		Houses	Blocks	
El Centro	643	1872	***2 Ovillantas***	12	3	24
			Ovitrap*	12		12
La Esperanza	402	1530	***2 Ovillantas***	12	3	24
			Ovitrap*	12		12
El Porvenir	477	1528	***2 Ovillantas***	12	3	24
			Ovitrap*	12		12
San Miguel	346	1412	***2 Ovillantas***	12	3	24
			Ovitrap*	12		12
El Triunfo	160	698	***2 Ovillantas***	12	3	24
			Ovitrap*	12		12
La Pista	116	519	***2 Ovillantas***	12	3	24
			Ovitrap*	12		12
La Hojita Verde	60	229	***2 Ovillantas***	12	3	24
			Ovitrap*	12		12
**Subtotal**	**2204**	**7788**		**168**	**21**	**252**
Control sites (Ovitraps)			Type of ovitrap	Distribution		Total
Name	# Houses	Inhabitants		Houses	Blocks	
La Democracia	229	1001	***Monitoring*** (ovitrap)*	12	3	12
La Virgencita	270	1046		12	3	12
Lomas del Norte	534	1772		12	3	12
El Pescador	290	1319		12	3	12
La Unión	157	710		12	3	12
El Limón	106	569		12	3	12
Santa Cruz	92	349		12	3	12
**Subtotal**	**1678**	**6766**		**84**	**21**	**84**
**Total**	**3882**	**14554**		**252**	**42**	**336**

* Monitoring with ovitraps model: Ministry de Health, México. Program of Control of Dengue Vigilance, Prevention y Control. CENAPRECE. Anexo 3

Once the ovillantas and ovitraps were installed in the different households, the health workers started cleaning the ovillantas in the presence of household members and, later on, as interest increased, household members themselves were responsible for cleaning the ovillantas or ovitraps, in the presence of health workers. At the beginning of our project, the joint care and egg collection was carried out once a week, on a day pre-established with the community. During the high season (June-October), it was necessary to monitor and clean the ovillantas and ovitraps twice a week, in order to avoid the production of mosquitoes due to the rapid development of the larvae during high temperatures. The pellon papers containing the eggs were removed and given to the health workers, and new clean pieces of paper were installed. The papers with the eggs were taken to the laboratory of the local health unit by the health workers, and counted there by specialized personnel.

At the beginning of the project we started with three different types of mosquito traps: an ecological ovillanta with *AE*-Lure, an ecological ovillanta with Malaria-Lure, and the standard ovitrap with water. But shortly after initiation, we discovered Malaria-Lure to be ineffective for malaria-parasite carrying *Anopheles* mosquitoes. Therefore, by week 4 we changed all ecological ovillantas to include AE-Lure only.

We measured mosquito control by the mean number of eggs counted in ecological ovillantas versus standard ovitraps during 31 collections over 10 months between Feb and Nov 2015. Egg counting was undertaken once a week.

**c) Community engagement.** Through training, our intersectoral multidisciplinary approach aimed to improve and sustain the technical competencies of health workers. Through the modification of the ovitrap, we were sensitive to cost, ecological concerns, and local applicability. For both of these integrated interventions to work, community involvement was required. Therefore, we simultaneously undertook community engagement activities, recognising the importance of sustained social participation by both community members and health workers.

We developed strategies to engage the main representatives of the community, in order to motivate participation in the mosquito control programme. We focused on education of the community (in addition to health workers) about the mosquito cycle, sustainable strategies for keeping homes and gardens clean and unattractive to mosquitoes, the implementation and maintenance of mosquito traps, and collaboration between community and health sectors to collectively manage vector control and prevention of mosquito-borne disease. Specifically, community members were taught how to safely keep the ovillantas or ovitraps, including how to clean the traps, change the pellon paper and hand the pellon paper to the health workers. They were incentivised by the education they received on how to reduce the amount of mosquitoes and thus keep themselves and the community healthy. Another strong incentive was the absence of fogging, thus no pesticides in their neighbourhood, unless they did not care for the ovillantas properly and there were too many mosquitoes.

We measured social participation by surveying community member and health worker perceptions, knowledge and participation in mosquito control using qualitative methods. (see Annex 3 for fuller details of the evaluation of social participation). Focus groups were undertaken in eight neighbourhoods, four from the study site and four from the control site. Within each neighbourhood, one person from 24 randomly selected households (12 households with either ovillantas or ovitraps and 12 households that did not have either, but were located within the neighbourhoods of the study) was invited to participate in a focus group. Of the sixteen focus groups, the number of participants ranged from three to eight household members each.

In addition, qualitative interviews were conducted with three health workers who undertook the training and participated in the mosquito control programme, and one programme manager. Evaluation of social participation and analysis of the qualitative data was carried out at the beginning (February 2015) during the setup of the ovillantas and ovitraps in each household, in order to establish a base level for evaluation. This was followed by a second interview at the end of the project (December 2015). The interviews were carried out following the protocol validated by the ETV/MSPAS representative and were conducted by an experienced researcher independent from the other two components of the project (ovillanta study, web-based training) (MB).

**Data analysis.** We analysed quantitative variables (egg count) and categorical variables (area, type of trap) for their frequency of distribution, measuring the central tendency with a graphical representation and distribution tests. The software package Stata13 ^®^ was used to carry out the t-tests, which analyze the difference between two means and the dispersion of the scores (see Annex 4).

With respect to the qualitative data, we undertook an analysis per topic to understand perceptions, social participation, usefulness of strategies for control and prevention of the diseases, negative and positive factors of the control programme, and recommendations for future strategies.

**Entomological poll.** A survey of potential breeding sites was conducted in each house of the intervention neighbourhoods, where the ovillantas were placed. Each container capable of accumulating water (drums, tires, cans, etc.) was analysed and larvae/pupae were counted if present. A percentage of each type of container that was positive was established compared to the total number of containers found (see Annex 5).

## Results

**a) Web-based training of health workers in vector control.** Twenty five health workers subscribed to the web-based training programme (of 35 eligible). Most had obtained elementary school level education (67%); the remainder had achieved junior high school level (18%), high school (10%), or university undergraduate level (5%). Following the web-based training (comprised of online lectures, case studies, virtual discussions and homework) teams of students worked on a proposed ecological intervention for the prevention and control of the vector mosquito in Sayaxche using a manual validated by the academic unit of the INSP ( Annex 1) and were evaluated (directly by the INSP evaluation personnel, independently from the project’s participants). Eighty percent of the students were accredited, obtaining between 75 and 95 points out of 100, and receiving a certification issued by the INSP.

The perceptions and evaluations of the course by the students and program coordinators were remarkably positive, recommending that the course be implemented in all health units across Guatemala. Nevertheless, several difficulties were reported by the students. The most important was the difficulty to access computer equipment and the lack of training on the use of computers with internet in remote places. Some students complained about the excess of homework and the little time allocated for it. However, it was a general consensus that the applied benefits generated by the learning process were immediately felt in the technical field work, making the health workers more reassured.

**b) Ecological ovillanta.** A total of 84 households (from 2204 in the area) were the focus of study within the 7 study site neighbourhoods. All 84 households that agreed to participate in our study were allocated to the study intervention (2 ecological ovillantas and 1 standard ovitrap). Intervention households were no more than 50 metres apart (in three continuous blocks), which we felt gave sufficient coverage because mosquitoes tend to fly up to 500 m around looking for blood or an oviposition site ^24^.

Ovillantas were set up on Feb 8, 2015; the monitoring ovitraps were installed only on April 12, 2015. All of the systems, ovillantas and control ovitraps, were monitored and cleaned by the health workers on a weekly basis, with community members present. The health workers filtered and recycled the attractant solution, counting and eliminating any eggs deposited on the landing strips. One interruption of this weekly schedule took place, for 5 weeks in August and September, due to labour and political problems within the Ministry of Health in Guatemala.

The mean weekly egg count was higher in neighbourhoods with ovillantas with a mean of 19.26334 (SE 0.4707; 95% CI: 18.34056, 20.18613) than at the control sites using standard ovitraps, with a mean of 13.2787 (SE 0.8249; 95% CI: 11.66214, 14.89748). The difference was statistically significant (t= 5.2577; *p*< 0.05).

**Figure 2.**
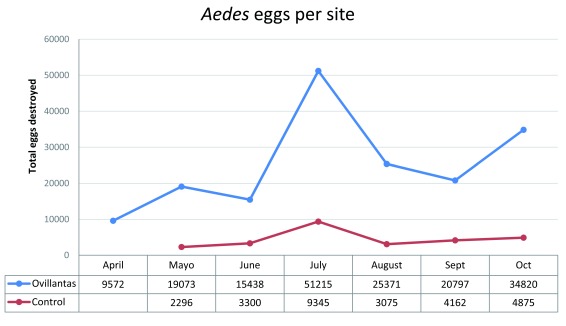
Monthly total eggs destroyed in intervention sites (blue) and control sites (orange). Left: Total eggs counted per site. Right: Eggs per site and per device (intervention/blue)/(control/orange). Total at intervention sites = 181,336 total *Aedes eggs*, Total at control sites = 27,053 total *Aedes* eggs.

Figure 2-left, shows the total amount of *Aedes* eggs collected and destroyed per month at the study sites (ovillantas/blue) (176,286 eggs over 10 months) and at the control site (ovitraps/orange) (27,053 eggs).

**Figure 3.**
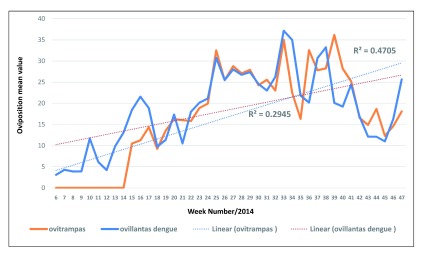
Weekly oviposition mean value in intervention sites (ovillantas-orange) and control sites (standard ovitraps-blue).

Figure 3 shows the mean value between the intervention sites (ovillantas) and control sites (standard ovitraps), during the 42 weeks of the study.

However, within the study site households, there were no statistically significant differences in the amount of *Aedes* eggs collected from ecological ovillantas and standard ovitraps over the 42 week study. We believe this may be because mosquitoes leave a natural pheromone with each egg each time they oviposit, thus the recycling may have concentrated the natural pheromone (the initial solution was not discarded, but instead new solution was added). Understanding this further will be the focus of a future study.

We also observed that independent of the data obtained per neighbourhood, the amount of *Aedes* eggs collected was greater on standard ovitraps (mean= 20. 514, SE 0.6181; 95% CI: 19.30213, 21.7254) when compared to ovillantas at the same site (mean= 15.854, SE 0.55027; 95% CI: 14.77511, 16.93254), with a statistically significant difference of t= -5.581 ( *p*< 0.05). Furthermore, we observed that standard ovitraps at the same sites as ovillantas (i.e., study sites) (mean= 23.985, SE 0.8103; 95% CI: 22.396, 25.574) collected more *Aedes* eggs than standard ovitraps at the control sites (no ovillantas present) (mean= 13.279, SE 0.824; 95% CI: 11.622, 14.89). We speculate that there may be a synergistic attraction effect when there are ovillantas present in close proximity to standard ovitraps, which could explain why the cluster of ovillantas/ovitraps produced better results per unit than an ovitrap alone ( Figure 2).

The observed amount of *Aedes* eggs differed within neighbourhood. There was also the competition of the ovillantas/ovitraps against natural breeding places within a given house, based upon an entomological poll we conducted ( Table 2). Among the study neighbourhoods, El Centro stands out as the site with highest egg count across the study period (mean= 47.23; 95% CI: 43.12, 51.34), and major breeding container diversity, with 127 different types (drums: 16.26%, laundry water basin: 21.95%, tubs: 9.76% and other smaller containers: 21.95%, and from each one of the container types: 40%, 37.04%, 16.97% and 22,22%, respectively presented a positive larval/pupae count). El Centro produced 23.58% of the total *Aedes* larvae found among all the study sites. In the neighbourhood La Esperanza (mean= 41.22) an average of 7.79% of the whole containers contained larvae/pupae. While the same were found among the laundry water basin and buckets/canisters in 12.50% of each type of container. In the neighbourhood of San Miguel 50% of the tires found contained larvae/pupae, followed by 37.50% of laundry water basins. In contrast the neighbourhoods of La Pista and Hojita Verde showed the lowest levels of larvae production among the inspected containers with 4.55% (mean= 4.05) and 4.44% (mean= 0.14), respectively. In both of these neighbourhoods the drums were the main containers where larvae were found (21.05% and 16.67%, respectively).

**Table 2.** Entomological poll in neighbourhoods that were part of the study (ovillantas).

Type of container	Centro		La Esperanza		Hojita Verde		La Pista		El Porvenir		San Miguel	
	N (%)	(%) Larva/ Pupae	N (%)	(%) Larva/ Pupae	N (%)	(%) Larva/ Pupae	N (%)	(%) Larva/ Pupae	N (%)	(%) Larva/ Pupae	N (%)	(%) Larva/ Pupae
Drum	20 (16.26)	40.00	2 (2.60)	0.00	19 (21.59)	21.05	18 (20.00)	16.67	0	0.00	16 (12.50)	18.75
Tires	7 (5.69)	0.00	4 (5.19)	0.00	0	0.00	2 (2.22)	0.00	2 (2.22)	0.00	8 (6.25)	50.00
Laundry basin	27 (21.95)	37.04	24 (31.17)	12.50	21 (23.86)	0.00	22 (24.44)	4.55	4 (31.08)	17.39	24 (18.75)	37.50
Cans and buckets	20 (16.26)	5.00	16 (20.78)	12.50	24 (27.27)	0.00	23 (25.56)	0.00	20 (27.03)	15.00	24 (18.75)	8.33
Tubs	12 (9.76)	16.67	7 (9.09)	0.00	0	0.00	0	0.00	5 (6.76)	0.00	24 (18.75)	0.00
Toilets	10 (8.13)	0.00	0	0.00	0	0.00	1 (1.11)	0.00	0	0.00	8 (6.25)	0.00
Small various	27 (21.95)	22.22	24 (31.17)	4.17	24 (27.27)	0.00	24 (26.67)	0.00	24 (32.43)	4.17	24 (18.75)	0.00
Total	123 (100)	23.58	77 (100)	7.79	88 (100)	4.55	90 (100)	4.44	74 (100)	10.81	128 (100)	14.06

Source: Entomological Poll of the Program in Sayaxche based on the normativity of the Ministry of Health, Mexico (CENAPRECE). N=Number of each water containers with larvae/pupae.

We were unable to conduct definitive viral identification tests because the technology does not exist, or is inaccessible in these remote locations, and was planed only for Phase II of the project. The low numbers among the official reports ( Table 3) show the limitations, in several communities around Sayaxche, of establishing a surveillance of confirmed or suspected cases. Cases are diagnosed only in extreme clinical conditions of fever, rash, etc.

**Table 3.** Dengue cases in the Sayaxche region (laboratory confirmed and extreme fever suspected as dengue). (data from: Laboratorio Nacional de Salud, Guatemala city, Guatemala).

Dengue cases/city (confirmed/ suspected)	El Naranjo	Las Cruces	La Libertad	Nueva Esperanza	Tierra Blanca	Sayaxche	Total
2014	-	1/0	1/0	1/0	-	1/0	4/0
2015	1/3	7/4	1/2	-	-	0*/1	9/10
2016	-	1/1	-	-	-	0/2	1/3
Population in 2000		8,246	14,972			14,554	

*There were 2 imported cases confirmed in Sayaxche.

***c) Social participation*.** Sixteen focus groups with household members were conducted, across eight neighbourhoods – four within the study site and four within the control site. Each group comprised three to eight participants (70% women), out of 12 people per focus group invited.

Taken together, several themes of sociocultural issues were identified from the focus groups, which can help guide future strategies geared to strengthen community participation in mosquito control:

1. Belief that the mosquitoes breed only in natural ponds, not in backyards.
2. Belief that cleaning of the house and garden are tasks of women only.
3. Belief that the Ministry of Health’s services are not efficient.
4. Preference for self-medication using local medicinal plants (e.g. Cat’s Claw ( *Uncaria tomentosa*) against dengue and chikungunya fever.
5. Very little information is provided to the public about the cause of illness and consequences.
6. Dependency of the community on public services to maintain the cleanliness of the streets.
7. Dependency of the community on public antimalarial control, without participation.
8. Low regard for the advantages of community participation.
9. Communication difficulties between some ethnic groups.
10. Low acceptance of new mosquito control methods and transmission prevention strategies, despite wanting less reliance on pesticides.

In terms of the ecological ovillanta as a form of mosquito control, community members provided several views. Overall, the recycling of the attractant solution was welcomed given the difficulty to obtain clean water in the region. There was satisfaction with the use of both the ovillantas and ovitraps as preventive methods and to reduce the overall number of adult mosquitoes. There was interest in knowing how many *Aedes* eggs were collected each week, and women in particular were keen to be more involved in the activities related to health and showed interest in knowing how the system worked and learning how to maintain it. Among those surveyed who did not have ovillantas, many showed interest in having them installed at their households. In general, the impression was that the ovillantas were effective in reducing adult mosquitoes.

Among the health workers and manager interviewed, the training programme was said to motivate and strengthen their social and technical field work. Financial resources, vehicles, fuel and personnel were said to be scarce. They requested more research in the field, so they could further learn. They expressed concern that implementation of any new strategies be sustained.

## Discussion

We found our three-component integrated intervention to have proved beneficial to this remote community at risk of mosquito-borne diseases such as dengue, chikungunya, and Zika. The combination of health worker training, low-cost ecological ovillantas, and community engagement ensured the project met local needs and fostered collaboration and participation of the community, which can help improve sustainability.

The integrated involvement and web-based certification of local health workers strengthened expertise in the area, and has generated evidence of ecosystem alternatives against *Aedes* mosquitoes.

The ecological ovillantas, made out of recycled material (garbage that when left in the field increases the potential of creating breeding sites), proved positive in the capture of *Aedes* eggs and possibly on the reduction of the adult mosquito populations, more work needs to be performed, using adult traps, to confirm this. A remarkable acceptance and willingness to participate was observed, not only from the community but also from health workers who monitored its implementation. This was bolstered by community members learning and observing the early biological life stages of the vector, as well as observing the number of *Aedes* eggs collected in their households, which permitted them to relate those to the presence and quantities of adult mosquitoes potentially produced. It is possible that there may be a synergistic effect between the motivation to participate in the control of mosquitoes, using the innovative strategy of the ovillantas, and the complementary actions needed to maintain an orderly and clean house and backyard. This serves to avoid the creation of artificial breeding sites for the *Ae aegypti* mosquito and the transmission of viral infections.

Interestingly, a recent report by Ayres ^25^ from the Brazilian Oswaldo Cruz Foundation described that species of mosquitoes other than *Aedes* can be infected with the Zika virus, including *Culex* spp. mosquitoes, in laboratory settings. Recent reporting from Brazil has also affirmed concerns that infection with Zika virus of *Culex* mosquitoes is vastly more common than *Aedes* and is being overlooked in the prevention and control efforts ^26^. So while the contribution of these other species to Zika transmission has not as yet been firmly established, we anticipate that our ovillanta approach could potentially be used to reduce populations of either species. For example, *Culex restuans/pipiens* mosquitoes were collected in the first Canadian study using a modified ovitrap. In the summer of 2007, approximately 3.2 million eggs (in rafts) were counted and destroyed in the city of Sudbury, Canada in a 90-day study, using 150 modified ovitraps, resulting in a 90% reduction of the adult *Culex* spp. population within the study sites (unpublished results Ulibarri *et al*.). The only requirement to attract a different species, or to have an effect on both *Aedes* and *Culex* mosquitoes in the region, is a change of the attractant solution used in the ovillanta, or the set up of a second ovillanta with different attractant solution – the equipment stays the same.

In general, the level of knowledge that community members and health workers held about different viral infections was almost non-existent and, at times, wrong. Clearly there is a need to carry out novel strategies aimed at gaining and maintaining the attention of the community; traditional recommendations provided by health workers tend to bore them, they said. More dynamism is necessary, especially with children. In addition, it is important to include the active participation of infected patients with health centres to avoid further mosquito infection. In this way, infected symptomatic cases can be properly recorded and monitored, and the patient can be followed accordingly.

The participation of the population in vector-borne disease prevention and control is an area that requires more effort and attention. One aspect requiring sensitivity is the clear extent of individualism within communities in this area of Guatemala, and the evident conflicts among different ethnic groups, mainly around culture and language. These work against social cohesion and participation. All the government groups responsible for ensuring health and safety require coordination and collaboration, including reducing the number of abandoned lots where mosquitoes breed. It would be beneficial for the government to apply an ecosystem approach ^27^ for the communal benefit and to establish a mechanism for people not to throw garbage on the streets and learn to recycle the same properly.

There were several limitations to our study.

Our project was not able to consider the inclusion of the epidemiologic impact of the methodology on viral transmission using sequential polls of seroprevalence. However, the intensity of dengue transmission while the project developed was higher in cities close to Sayaxche, such as Las Cruces and La Libertad. During our study period, the reported incidence of dengue in 2015 for La Libertad, Las Cruces and Sayaxche was 3.33, 10.91 and 2.06 per 10,000 pop., respectively. This was also a direct observation of the Director of the Program in Sayaxche with respect to the buffering effect possibly associated with the intervention using the ovillantas. The Pan American Health Organization (PAHO) reports 18,058 probable cases (1,228 laboratory-confirmed cases) across Guatemala in 2015 ^28^ and 1,179 probable cases up to week 11 of 2016. We ask ourselves, how has the city of Sayaxche been spared this national burden? The confirmed incidence in La Libertad was 5.29 times higher than the ones registered in Sayaxche during the same year. The effectiveness of the ovillanta intervention on the reduction of dengue virus transmission within the city of Sayaxche could be based on the fact that zero autochthonous cases were reported, and only three imported cases were confirmed (unpublished data, Area de Salud Peten-Suroccidental). We remain cautious and will continue to monitor the epidemiology closely. 

Second, while we believe the neighbourhoods and households shared the same or similar features in terms of sociodemographics, climate, and others, we did not specifically measure these variables. And although we systematically monitored the presence of backyard containers with larvae or potential water collection (in part through the entomological poll), their presence was not documented nor measured in households that may also have served as mosquito breeding places (i.e., tubs, drums, etc. that filled with rain water).

Third, we did not implement and complete the health worker training prior to initiating the second and third components of the study. The web-based training for health workers was provided simultaneously, showing that the trained personnel gained a better understanding of the vector control process and a better transfer of information to the community. All of the health workers involved in installing and monitoring the ovillantas undertook the web-based training, and only one failed to be accredited. We believe that early training of the personnel, prior to interacting with the community, might have produced better results. The fact that the community were already acquainted with most of the health workers enabled their acceptance of the community engagement activities and of the ovillantas study.

Nonetheless, our project provides evidence for a promising alternative to harmful pesticides and standard ovitraps at a time when the threat of viral outbreaks is increasing. By incorporating ecology and community-oriented elements, this alternative has the potential to be effectively scaled-up and be sustainable.

## Data availability

The data referenced by this article are under copyright with the following copyright statement: Copyright: © 2017 Ulibarri G et al.

Data associated with the article are available under the terms of the Creative Commons Zero "No rights reserved" data waiver (CC0 1.0 Public domain dedication). 

## Supplementary material (Spanish-language)

Annex 1. Intensive course to strengthen the vigilance, prevention and control of dengue transmission, an ecosystematic approach. (Curso Intensivo para el fortalecimiento de la vigilancia, prevencion y control del dengue con enfoque ecosistemico, 16 pages).

.

Annex 2. Competence and curricular program of the Course offered to the tactical operations personnel in Sayaxche, Peten, Guatemala. (Competencia y MOdulos Curriculares de; Curso para el Personal Tactico Operativo de Sayaxche, Peten, Guatemala, 1 page, small print).

.

Annex 3. Evaluation of the Project: Integral Participation and use of ovillantas in the prevention and control of Malaria and dengue in Sayaxche.) (Evaluación del proyecto *Participación integral y ovitrampas en la prevención y control del vector de la Malaria y Dengue en Sayaxché,* 21 pages).

.

Annex 4. Database Sayaxche 2015. The raw data of *Aedes* spp. egg collection during the study in Sayaxche, Guatemala during 10 months of 2015.

.

Annex 5. Raw data of the Enthomological Poll.
