# Supplementary material for: Preliminary results on the control of Aedes spp. in a remote Guatemalan community vulnerable to dengue, chikungunya and Zika virus: community participation and use of low-cost ecological ovillantas for mosquito control
Source: F1000Res. 2017 Feb 22;5:598. Originally published 2016 Apr 7. [Version 3] doi: 10.12688/f1000research.8461.3 (PMC5225411; doi:10.12688/f1000research.8461.3)
Supplement: Supplementary file 1 [file f1000research-5-11650-s0000.tgz › 4c8b1226-1cf2-4b2d-bc36-96d4ecebbe55.pdf]

Formato C

INSTITUTO NACIONAL DE SALUD PÚBLICA
COLEGIO DE PROFESORES:

PROGRAMA DE EDUCACIÓN CONTINUA

UNIDAD DIDÁCTICA O MODULO:

Curso Intensivo para el Fortalecimiento de la Vigilancia, Prevención y Control del Dengue con Enfoque Ecosistemico.

SEDE: **Sayaxche, Peten. Unidad de Salud Suroccidental del Peten. Guatemala CA.**

FECHAS (jueves y viernes de cada semana)
INICIO: Febrero del 2015
TÉRMINO: Marzo del 2015

Autor del diseño de Unidad didáctica o módulo	Angel Francisco Betanzos Reyes	
Equipo docente	Nombre completo	Horas
Profesor titular:	Angel Francisco Betanzos Reyes	5
Profesor adjunto:		15
Profesores invitados:		10
Profesores invitados:		10

Presentación:

Este curso que ofrece el Instituto Nacional de Salud Pública (INSP) a través de su modalidad presencial, está sustentado en el modelo pedagógico por competencias adoptado institucionalmente y ofrecen la posibilidad de adquirir y desarrollar conocimientos, habilidades, actitudes y valores para un desempeño técnico y profesional acorde a las necesidades de la diversidad de contextos dinámicos de responsabilidad. En este sentido, tiene el propósito de desarrollar y potencializar capacidades técnicas operativas en los responsables del programa de prevención de dengue en Sayaxche, que les permita abordar con efectividad y eficiencia un programa preventivo

Su preparación se basa en las necesidades y competencias específicas para el personal operativo de salud que tiene contacto con pacientes que cursan con dengue así como población en riesgo. Se apega a los lineamientos establecidos por la OMS y por la normatividad vigente en México.

Mediante un proceso dinámico de enseñanza-aprendizaje teórico y practico sobre el manejo integral de la prevención y control de dengue, en este proceso se aplican estrategias a fin de fortalecer el aprendizaje con clases específicas, tareas dirigidas, revisión de literatura y análisis de información local y nacional para la vigilancia epidemiológica entre otras dinámicas, siempre con participación activa de los alumnos.

La organización de este curso permitirá al alumno conocer la dinámica de la enfermedad para traducirlo y adecuarlo a su contexto local, identificar áreas y grupos vulnerables o de riesgo, dirigir y aplicar el adecuado y oportuno manejo de la prevención y control del dengue integrando a la población en el autocuidado de la salud.

Competencias profesionales.

1. Conocer y monitorear la dinámica de transmisión de la enfermedad en su área de salud
2. Realizar análisis micro y macro regional de la dinámica de transmisión del dengue
3. Diseñar y conducir acciones para la prevención y control del dengue con la poblaciones con baja endemia
4. Planificar conducir acciones para el control del dengue en poblaciones con brotes
5. Desarrollar habilidades para el trabajo en equipo integrando la participación de la comunidad en la prevención y control de dengue en diferentes escenarios y contextos

Competencias específicas.

1. Explicar las condiciones epidemiológicas del dengue mundial, continental, nacional y local
2. Interpretar los componentes de la transmisión del dengue.
3. Identificar los factores de riesgo y determinantes en la transmisión global, estatal y local
4. Realizar, analizar y coordinar los procesos de diagnóstico por laboratorio, vigilancia y prevención

integral de áreas de riesgo (vulnerables y receptivas) en el contexto estatal

5. Reconocer los elementos, procesos y utilidad de la vigilancia integral (epidemiológica y entomológica) y establecer mecanismos para el flujo efectivo de la información a diferentes niveles gerenciales y operativos del programa.
6. Implementar acciones de vigilancia, prevención y control del dengue con enfoque ecosistemico en el nivel local y Departamental , utilizando estrategias y herramientas adecuadas, que incluyen la convergencia y gestión de conocimientos, habilidades, actitud y trabajo en equipo en implementación y desarrollo operativo con alto impacto.
7. Explicar los componentes del enfoque de Ecosalud y los beneficios que aporta al manejo de las ETV (Dengue).
8. Identificar áreas de oportunidad para incorporar acciones para la vigilancia, prevención y control del dengue (ETV´s) con enfoque en Ecosalud.
9. Informar y sensibilizar a la población sobre los cuidados para prevenir y evitar la transmisión del dengue personal, familiar y en su entorno colectivo

Competencias transversales.

1. Aplicación de habilidades y estrategias para desarrollo en campo de la participación y colaboración de la población
2. Gestión y cultura de integración y trabajo en equipo con interacción con diversos sectores y programas de salud.
3. Ejercer acciones operativas proactivas, sostenibles y alto impacto en el control sostenido del dengue
4. Analizar información científica y/o normativa, desarrollando un conocimiento y experiencia en la interpretación y solución del problema del dengue
5. Practicar la adecuada toma de decisiones en el manejo integral del dengue en las áreas de riesgo

Contenido temático:

Modulo I. Epidemiología de Dengue

1. Situación global, continental, nacional, estatal y local del dengue.
2. Transmisión del dengue. Componentes (ciclo biológico del dengue, historia natural), interacciones, patrones-variantes)
3. Agente etiológico, descripción del DENV
4. Factores de riesgo y determinantes del dengue.
5. Notificación epidemiológica del dengue

6. Diagnóstico y tratamiento oportuno

Modulo II. Vigilancia entomológica en fase larvaria y adulta
Indicadores entomológicos

1. Acciones para la vigilancia entomológica en áreas sin transmisión
2. La vigilancia entomológica en áreas de riesgo sin transmisión activa
3. Vigilancia entomológica en áreas de riesgo con transmisión activa de la enfermedad
4. Monitoreo entomológico en situaciones de crisis

Módulo III. Participación Comunitaria, sitios de concentración poblacional y áreas de riesgo libres de criaderos

5. Acercamiento comunitario.
6. Planeación comunitaria.
7. Reunión con grupos sociales. Transdiciplina e intersectorialidad.
8. Supervisión y evaluación de la participación comunitaria
9. Mecanismos de respuesta basados en la información. Atención de riesgos y respuesta a brote con la participación de la comunidad
10. Interfase Comunidad-Programa (Participación social y comunitaria)

Modulo IV. Control integrado del vector

1. Practicas efectivas. Medidas químicas, físicas y biológicas
    a. Estratificación del riesgo
    b. Control y reducción de la abundancia del vector
        1. Control de criaderos
        2. Rociado intradomiciliar
        3. Mosquiteros impregnados con insecticidas
        4. Rociado espacial
        5. Control integrado e vectores.
2. Enfoque Ecosistemico en la VPC del dengue.
    (i) Generalidad

(ii) Principios

(iii) Áreas de oportunidades de implementación del enfoque ecosistemico

3. Calidad operativa. Gestión, organización, supervisión y cobertura efectiva.

Metodología de enseñanza-aprendizaje:

Para el logro de las actividades diseñadas en el curso se han tenido en cuenta estrategias que faciliten la interacción, la generación de un pensamiento crítico y creativo para el desarrollo de las competencias que deben desarrollar los integrantes del programa operativo. La lógica del curso está prevista para que los participen desarrollen esta competencias. Es por ello que los asistentes serán los protagonistas del proceso de aprendizaje y los docentes cumplirán las funciones de facilitadores contando con su experiencia y apoyados en los materiales a disposición, utilizando las herramientas de la comunicación y el intercambio presencial con el profesor.

Las actividades se han diseñado teniendo en cuenta las diferentes etapas del ciclo de aprendizaje las que transitan desde la activación del conocimiento, la práctica y su transferencia lo que permitirá llegar a los resultados esperados.

De esta manera el desarrollo de los temas serán se llevaran a cabo en una dinámica teórica y práctica (presentaciones, ejercicios en clase, y seguimiento para una trabajo final, en lo posible, sobre un plan integral de prevención y control sostenido del dengue con participación comunitaria.

Se llevará a cabo un curso intensivo, que se adecuará al perfil de los alumnos y al tiempo considerado de 8 días presenciales, distribuidos en cuatro semanas (jueves y viernes de cada semana) a partir del 3 y finalizando el 25 de octubre del 2013

En este curso se establecerán varias estrategias de trabajo para abordar los contenidos propuestos:

1. Consulta de materiales: al inicio de cada modulo se proporcionaran lecturas para análisis grupal de la información mas reciente para la prevención y control del dengue. Así mismo se proporcionaran presentaciones a fin de que el estudiante pueda revisar, analizar, y reflexionar sobre el tema.
2. Tareas: Con la finalidad de analizar, aplicar y reflexionar sobre el tema revisado y solo después de los primeros 3 módulos se dejaran tareas dirigidas, mismas que el alumno entregara en el módulo siguiente. Estas actividades pueden ser: control de lectura, ejercicios prácticos o resolución de casos
3. Exámenes: se realizará un examen diagnóstico y un examen final a fin de evaluar los temas vistos en el curso
4. Sesiones presenciales: Actividad donde el profesor expondrá los temas actuales y de importancia para fortalecer el conocimiento de los participantes y permitirá la retroalimentación directa en las dudas que se generen durante el curso.

MATERIALES DE TRABAJO. (Insumos de trabajo académico y de fortalecimiento técnico-operativo**)**

- Bases de datos e informes históricos (últimos 10 años o el disponible) de casos confirmados autóctonas e importados, intervenciones de control (tipo, frecuencia y cobertura),

- Mapas y croquis de comunidades (hidroentomológicos) y áreas estatales de rutas migratorias, carreteras y áreas identificadas con perfil entomológico y de riesgo

- Recursos. Infraestructura, personal, equipos, insumos, laboratorios (ubicación y capacidad operativa).

- Bibliografía básica. Referida en el siguiente inciso.

Criterios de Evaluación:

El curso utilizara los diferentes tipos de evaluación: diagnóstico, formativa y sumativa
Evaluación diagnóstico: se utilizara un pretest
Evaluación formativa: aportaciones significativas en clases y entrega a tiempo de las asignaciones.
Evaluación Sumativa:

ASPECTOS	PONDERACION (100%)
Puntualidad y participación en clases	10%
Trabajos en equipo<br>• Mapas conceptuales, esquemas, etc. (30%)<br>• Discusión en plenarias (30%)	40%
Tareas (3)	30%
Evaluación final (aprobatoria <8)	20%
Total	100%

Bibliografía.

Básica:

- DENGUE, Guías para el diagnóstico, tratamiento, prevención y control. OMS-OPS, 2009.
- Manual para la vigilancia, diagnóstico, prevención y control del dengue. Secretaria de Salud México. 2006.
- DENGUE. Guías de atención para enfermos en la región de las Américas. OPS, 2010
- Programa de Acción específico 2007-2012, dengue. Secretaría de Salud México, 2007.
- NOM 017 SSA2-2012 para la Vigilancia Epidemiológica
- NOM032 SSA2-2010 para la Vigilancia Epidemiológica, Prevención y Control de las Enfermedades Transmitidas por Vector.
- Guía Metodológica para Control Larvario. Secretaría de Salud, CENAPRECE. México 2012
- Guía Patio Limpio y Cuidado del Agua Almacenada. Secretaría de Salud, CENAPRECE. México 2012
- Guía Entomológica fase larvaria. Secretaría de Salud, CENAPRECE. México 2012
- Guía Nebulización Espacial. Secretaría de Salud, CENAPRECE. México 2012
- Guía Pruebas biológicas. Secretaría de Salud, CENAPRECE. México 2012

- Guía Rociado residual intradomiciliar. Secretaría de Salud, CENAPRECE. México 2012
- Guía Vigilancia Entomológica Ovitrampas. Secretaría de Salud, CENAPRECE. México 2012

Complementaria:

- **Base de datos Cochrane de revisiones sistemáticas**
  http://www.cochrane.de/default.html
- **CDC**
  http://www.cdc.gov/spanish/
- **CENAPRECE**
  www.cenaprece.salud.gob.mx
- **Otros recursos importantes**
  http://www.wordreference.com/es/ : Traducción, sinónimos, antónimos en inglés, francés y español
  http://thesaurus.com/: Traducciones
  http://www.rae.es/rae.html: Diccionario virtual de la real academia española de la lengua.

Cápsulas bibliográficas:

**Profesor titular**	
**Nombre completo**	**Dr. Ángel Francisco Betanzos Reyes**
**Nacionalidad (en caso de ser extranjero)**	
**Nivel académico, área de formación e institución donde obtuvo último grado de estudios**	**Doctorado en Ciencias en Salud Pública (Epidemiología)**
**Establecimiento o dependencia de procedencia**	**INSP**
**Cargo y puesto**	**Médico especialista**
**Área (s) de interés**	**Salud Pública y ETV´s**
**E-mail**	**abetanzos@insp.mx**

**Profesor adjunto**	
**Nombre completo**	**Dr. Nohemí Colín Soto**
**Nacionalidad (en caso de ser extranjero)**	
**Nivel académico, área de formación e institución donde obtuvo último grado de estudios**	**Maestría en Epidemiología**
**Establecimiento o dependencia de procedencia**	**SSA/CENAPRECE. Programa de PC Dengue**
**Cargo y puesto**	**Médico especialista**
**Área (s) de interés**	**Salud Pública**
**E-mail**	**nohemicolin@yahoo.com.mx**

**Profesor adjunto (tutor)**	
**Nombre completo**	**Lic TS. Rogelio Archundia González**
**Nacionalidad (en caso de ser extranjero)**	
**Nivel académico, área de formación e institución donde obtuvo último grado de estudios**	**Licenciatura en Trabajo Social**
**Establecimiento o dependencia de procedencia**	**CENAPRECE**
**Cargo y puesto**	**Supervisor Profesional de Trabajo Social en Área Médica**
**Área (s) de interés**	**Salud Pública**

**E-mail**	**rogarchundia@hotmail.com**

**Profesor adjunto (tutor)**	
**Nombre completo**	**Biol. Víctor Salazar Bueyes**
**Nacionalidad (en caso de ser extranjero)**	
**Nivel académico, área de formación e institución donde obtuvo último grado de estudios**	**Estudiante de Maestría en Ciencias INSP**
**Establecimiento o dependencia de procedencia**	**CENAPRECE/ INSP**
**Cargo y puesto**	**Biólogo de la Dirección de Enfermedades Transmitidas por Vector**
**Área (s) de interés**	**Salud Pública**
**E-mail**	

Programa detallado de la unidad didáctica

**3 de Octubre 2013**<br><br>Apertura 1 Hora (presencial)	**Presentación y evaluación basal del curso. Análisis integral del dengue. 5 horas presenciales, 4 temas, 2 actividades**
Competencias	1. Explicar las condiciones epidemiológicas de la enfermedad mundial, continental, nacional y estatal.<br>2. Interpretar los componentes e interacciones de la transmisión del dengue.<br>3. Identificar los factores de riesgo y determinantes en la transmisión global, estatal y local
8.00- 09.00	INAUGURACION. . SECRETARIO DE SALUD O SU REPRESENTANTE

Fecha/Hora	Actividad	Modalidad	Materiales didácticos a utilizar	Evidencia de aprendizaje
**03/10/13**				
9.00 - 9.30	Examen diagnóstico	Trabajo individual	Formato impreso	
9.30 - 9.50	Situación del dengue mundial	Presentación.	Presentación.	Discusión en grupo, participación espontánea, lluvia de ideas
9.50 - 10.20	Situación Nacional del dengue	Presentación.	Presentación.	
10.20 - 10.40	Situación estatal del dengue	Presentación.	Presentación.	
10.40 - 11.20	Agente etiológico	Presentación.	Presentación.	
11.20 - 11.30	Receso	Presentación.	Presentación.	
11.30 - 12.30	Componentes de la transmisión	Presentación.	Presentación.	
12.30 - 13.30	Factores de riesgo y determinantes	Presentación	Presentación	
13.30 - 14.00	Discusión de la dinámica de transmisión	Trabajo en equipo	Impreso para Análisis de un brote	Discusión y compromisos con participación individual.

4 de octubre, 2013.<br><br>**Modulo I**	**Bases para la vigilancia epidemiológica del dengue en México. 5 horas presenciales. 2 temas, 2 actividades y 1 tarea**
Competencias	Conocer la metodología para la vigilancia epidemiológica del dengue en el país

Hora	Actividad	Modalidad	Materiales didácticos a utilizar	Evidencia de aprendizaje
09:00-12:00<br><br>3 horas	Diagnóstico clínico y por laboratorio del dengue<br><br>Actividades para la notificación	Presentación magistral<br><br>Discusión y participación individual	Presentación<br><br>Programa: Documento en Word con material impreso	Participación espontanea.<br><br>Discusión y participación individual.
12:00- 14:00<br><br>2 horas	Mesas de trabajo. Mapa estatal estratificando comunidades de riesgo epidemiológico	Organización de mesas de trabajo con discusión, preparación del mapa de riesgo y presentación en panel	Impreso. Mapa con ubicación de comunidades.	Dinámica de análisis por equipos de trabajo y discusión de resultados.

10 de octubre, 2013.<br><br>**Modulo II**	**Vigilancia entomológica. 5 horas presenciales. 4 presentaciones. 1 actividad**<br><br>• Vigilancia entomológica del vector en fase larvaria<br>• Vigilancia entomológica del vector en fase adulta
Competencias	Realizar, analizar y coordinar los procesos de monitoreo entomológico del vector en fase larvaria

Hora	Actividad	Modalidad	Materiales didácticos a utilizar	Evidencia de aprendizaje
09:00-10:00	Biología del vector	Presentación	Presentación	Participación espontanea.
10:00-11:00	Acciones para la vigilancia entomológica en áreas sin transmisión	Presentación	Presentación	Discusión y participación individual.
11:00-12:00	Acciones para la vigilancia entomológica en áreas de riesgo sin transmisión activa	Presentación	Presentación	
12:00-13:00	La vigilancia entomológica en áreas de riesgo con transmisión activa de la enfermedad	Presentación	Presentación	
13:00- 14:00	Mesas de trabajo.<br><br>Mapeo entomológico	Organización de mesas de trabajo con discusión de los riesgos entomológicos	Impreso mapa hidroentomológico.	Dinámica de análisis por equipos de trabajo y discusión de resultados.

11 de octubre, 2013.<br><br>**Modulo II**	**Vigilancia entomológica. 5 horas presenciales, 4 presentaciones, 1 actividad en clase y 1 tarea**<br><br>• Vigilancia entomológica del vector en fase larvaria<br>• Vigilancia entomológica del vector en fase adulta.
	Realizar, analizar y coordinar los procesos de monitoreo entomológico del vector en fase larvaria

Hora	Actividad	Modalidad	Materiales didácticos a utilizar	Evidencia de aprendizaje
09:00-12:00	Monitoreo entomológico en situaciones de crisis<br><br>Indicadores entomológicos<br><br>Organización de acciones entomológicas<br><br>Evaluación y supervisión de acciones entomológicas	Presentación<br><br>Discusión y participación individual	Presentación<br><br>Programa: Documento en Word con material impreso	Participación espontanea.<br><br>Discusión y participación individual.
12:00- 14:00	Taller grupal:<br><br>Elaboración de plan de trabajo a seguir en la localidad de acuerdo al mapeo entomológico obtenido	Organización de mesas de trabajo con discusión, preparación	Presentación docente y discusión grupal.	Dinámica de análisis por equipos de trabajo y discusión de resultados.

Formato C

17 de octubre., 2013.<br><br>**Modulo III**	**Patio Limpio y Cuidado del Agua Almacenada. 5 horas presenciales; 3 temas y 1 actividad.**<br><br>Etapa 1. Acercamiento comunitario<br><br>Etapa 2. Planeación comunitaria<br><br>Etapa 3. Reunión con grupos sociales<br><br>Etapa 4. Supervisión y evaluación
Competencias.	Reconocer la metodología empleada para lograr la participación comunitaria en la prevención y control de Dengue.

Hora	Actividad	Modalidad	Materiales didácticos a utilizar	Evidencia de aprendizaje
09:00-12:00<br><br>3 horas	Acercamiento comunitario.<br><br>Planeación comunitaria.	Presentación<br><br>Presentación	Guía de Patio Limpio y Cuidado del Agua Almacenada y discusión en grupo	Participación espontánea.<br><br>Discusión y participación individual.
12:00- 14:00<br><br>2 horas	Reunión con grupos sociales.<br><br>Integración de la intervención comunitaria	Presentación<br><br>Trabajo en equipo	Presentación docente y discusión en grupo<br><br>Impreso de un modelo de intervención	Análisis y discusión de resultados.<br><br>Lluvia de ideas

18 de octubre., 2013.<br><br>**Modulo III**	**Patio Limpio y Cuidado del Agua Almacenada. 5 horas presenciales. 3 presentaciones, 2 actividades y 1 tarea**<br><br>Sitios de Concentración Poblacional y Áreas de Riesgo**.** 4 horas presenciales; 2 temas y 2 actividades.
Competencia	Reconocer la forma de evaluar las actividades realizadas

Hora	Actividad	Modalidad	Materiales didácticos a utilizar	Evidencia de aprendizaje
09:00-10:00<br><br>2 horas	Supervisión y evaluación.	Presentación<br><br>Discusión y participación individual	Presentación Indicadores y formatos	Participación espontánea.<br><br>.
10:00- 12:00<br><br>1 horas	Sitios de Concentración Poblacional y Áreas de Riesgo**.**	Organización de mesas de trabajo con discusión, preparación del esquema	Presentación, Exposición de Ejemplos de Ambas Estrategias	Dinámica de análisis por equipos de trabajo y discusión de resultados.
12:00- 14:00<br><br>2 horas	Formatos utilizados fortalezas y debilidades de las estrategias presentadas y evaluación del modulo…	Organización de mesas de trabajo.	Presentación y discusión.	Dinámica de análisis por equipos de trabajo y discusión de resultados.

24 de octubre, 2013.<br><br>**Modulo IV**	**Control integrado del vector**5 horas presenciales. 7 presentaciones. .<br>• Practicas efectivas , medidas físicas, químicas y biológicas<br>• . Enfoque Ecosistemico en la Prevención y Control del Dengue
Competencia	1. Operar acciones para el control del vector<br>2. Explicar los componentes del enfoque de Ecosalud y los beneficios que aporta al manejo de las ETV (Dengue).<br>3. Identificar áreas de oportunidad para incorporar acciones para la vigilancia, prevención y control del dengue (ETV´s) con enfoque en Ecosalud.

Hora	Actividad	Modalidad	Materiales didácticos a utilizar	Evidencia de aprendizaje
09:00-12:00	Estratificación de riesgo<br><br>Control y reducción del vector<br><br>Control de criaderos<br><br>Rociado intradomiciliar<br><br>Uso de mosquiteros impregnados<br><br>Rociado espacial<br><br>Enfoque Ecosistemico en Dengue (ETV´s)	Presentación magistral<br><br>Discusión y participación individual	Presentación ptt<br><br>Programa: Documento en Word con material impreso	Participación espontanea.<br><br>Discusión y participación individual.
12:00- 14:00		Organización de mesas de trabajo (Mesa de Eliminación, Control, Riesgo: receptividad & vulnerabilidad) y construcción del mapa conceptual	Presentación docente y discusión de presentación magistral, material bibliográfico y discusión grupal.	Dinámica de análisis por equipos de trabajo y discusión de resultados.

25 de octubre, 2013.<br><br>**Modulo IV**	**Control integrado del vector.** 5 horas presenciales. 3 presentaciones.<br><br>• Practicas efectivas , medidas físicas, químicas y biológicas			
Competencia	1. Operar acciones para el control del vector<br>2. Explicar los componentes del enfoque de Ecosalud y los beneficios que aporta al manejo de las ETV (Dengue).<br>3. Identificar áreas de oportunidad para incorporar acciones para la vigilancia, prevención y control del dengue (ETV´s) con enfoque en Ecosalud.			
**Hora**	**Actividad**	**Modalidad**	**Materiales didácticos a utilizar**	**Evidencia de aprendizaje**
09:00-12:00	Estratégias de control efetivo<br><br>Enfoque ecosistémico (oportunidades y benefícios)<br><br>Control integral y enfoque ecosistémico<br><br>Calidad operativa organización, supervisión y cobertura efectiva<br><br>Evaluación de actividades de control (indicadores)	Presentación<br><br>Presentación<br><br>Presentación<br><br>Presentación	Presentación	Participación espontanea.<br><br>Discusión y participación individual.
12:00- 14:00	Construir un esquema estructurado sobre los elementos que describen las diferencias entre el enfoque tradicional y Ecosistemico en análisis del problema y respuesta en la vigilancia, prevención y control del dengue en Querétaro.	Organización de mesas de trabajo.	Presentación docente y discusión grupal.	Dinámica de análisis por equipos de trabajo y discusión de resultados.
14:00- 14:30	Evaluación final y clausura	Individual	Examen impreso	
