# Supplementary material for: Preliminary results on the control of Aedes spp. in a remote Guatemalan community vulnerable to dengue, chikungunya and Zika virus: community participation and use of low-cost ecological ovillantas for mosquito control
Source: F1000Res. 2017 Feb 22;5:598. Originally published 2016 Apr 7. [Version 3] doi: 10.12688/f1000research.8461.3 (PMC5225411; doi:10.12688/f1000research.8461.3)
Supplement: Supplementary file 2 [file f1000research-5-11650-s0001.tgz › fcd4b761-2276-4afa-99a6-33640d509814.pdf]

Anexo 2. Competencia y Módulos Curriculares del Curso para el Personal Táctico Operativo de Sayaxche, Peten. Guatemala

COMPETENCIAS			MODULOS TEMATICOS
**Generales**	**Específicos**	**Transversales**	
1.Conocer y monitorear la dinámica de transmisión de la enfermedad en su área de salud<br><br>2. Realizar análisis micro y macro regional de la dinámica de transmisión del dengue<br><br>3. Diseñar y conducir acciones para la prevención y control del dengue con la poblaciones con baja endemia<br><br>4. Planificar conducir acciones para el control del dengue en poblaciones con brotes<br><br>5. Desarrollar habilidades para el trabajo en equipo integrando la participación de la comunidad en la prevención y control de dengue en diferentes escenarios y contextos	-Explicar las condiciones epidemiológicas del dengue mundial, continental, nacional y local<br><br>- Interpretar los componentes de la transmisión del dengue.<br><br>- Identificar los factores de riesgo y determinantes en la transmisión global, estatal y local<br><br>- Realizar, analizar y coordinar los procesos de diagnóstico por laboratorio, vigilancia y prevención integral de áreas de riesgo (vulnerables y receptivas) en el contexto estatal<br><br>- Reconocer los elementos, procesos y utilidad de la vigilancia integral (epidemiológica y entomológica) y establecer mecanismos para el flujo efectivo de la información a diferentes niveles gerenciales y operativos del programa.<br><br>- Implementar acciones de vigilancia, prevención y control del dengue con enfoque ecosistemico en el nivel local y Departamental, utilizando estrategias y herramientas adecuadas, que incluyen la convergencia y gestión de conocimientos, habilidades, actitud y trabajo en equipo en implementación y desarrollo operativo con alto impacto.<br><br>- Explicar los componentes del enfoque de Ecosalud y los beneficios que aporta al manejo de las ETV (Dengue).<br><br>- Identificar áreas de oportunidad para incorporar acciones para la vigilancia, prevención y control del dengue (ETV´s) con enfoque en Ecosalud.<br><br>- Informar y sensibilizar a la población sobre los cuidados para prevenir y evitar la transmisión del dengue personal, familiar y en su entorno colectivo	- Aplicación de habilidades y estrategias para desarrollo en campo de la participación y colaboración de la población.<br><br>- Gestión y cultura de integración y trabajo en equipo con interacción con diversos sectores y programas de salud.<br><br>- Ejercer acciones operativas proactivas, sostenibles y alto impacto en el control sostenido del dengue<br><br>- Analizar información científica y/o normativa, desarrollando un conocimiento y experiencia en la interpretación y solución del problema del dengue<br><br>- Practicar la adecuada toma de decisiones en el manejo integral del dengue en las áreas de riesgo	**Modulo I. Epidemiología de Dengue**<br><br>1. Situación global, continental, nacional, estatal y local del dengue.<br>2. Transmisión del dengue. Componentes (ciclo biológico del dengue, historia natural), interacciones, patrones-variantes)<br>3. Agente etiológico, descripción del DENV<br>4. Factores de riesgo y determinantes del dengue.<br>5. Notificación epidemiológica del dengue<br>6. Diagnóstico y tratamiento oportuno<br><br>**Módulo II. Vigilancia entomológica en fase larvaria y adulta. Indicadores entomológicos**<br><br>1.Acciones para la vigilancia entomológica en áreas sin transmisión<br>2. La vigilancia entomológica en áreas de riesgo sin transmisión activa<br>3. Vigilancia entomológica en áreas de riesgo con transmisión activa de la enfermedad<br>4. Monitoreo entomológico en situaciones de crisis<br><br>**Módulo III. Participación Comunitaria, sitios de concentración poblacional y áreas de riesgo libres de criaderos**<br><br>1. Acercamiento comunitario.<br>2. Planeación comunitaria.<br>3. Reunión con grupos sociales. Transdisciplina e intersectorialidad.<br>4. Supervisión y evaluación de la participación comunitaria<br>5. Mecanismos de respuesta basados en la información. Atención de riesgos y respuesta a brote con la participación de la comunidad<br>6. Interfase Comunidad-Programa (Participación social y comunitaria)<br><br>**Módulo IV. Control integrado del vector**<br><br>1. Practicas efectivas. Medidas químicas, físicas y biológicas<br>2. Estratificación del riesgo<br>3. Control y reducción de la abundancia del vector (Control de criaderos, Rociado intradomiciliar, Mosquiteros impregnados con insecticidas, rociado espacial, control integrado e vectores).<br>4. Enfoque Ecosistemico en la VPC del dengue (Generalidad, Principios, áreas de oportunidades de implementación del enfoque ecosistemico)<br>5. Calidad operativa. Gestión, organización, supervisión y cobertura efectiva.
