# Supplementary material for: Preliminary results on the control of Aedes spp. in a remote Guatemalan community vulnerable to dengue, chikungunya and Zika virus: community participation and use of low-cost ecological ovillantas for mosquito control
Source: F1000Res. 2017 Feb 22;5:598. Originally published 2016 Apr 7. [Version 3] doi: 10.12688/f1000research.8461.3 (PMC5225411; doi:10.12688/f1000research.8461.3)
Supplement: Supplementary file 3 [file f1000research-5-11650-s0002.tgz › 676ea901-b029-4548-b170-678696aa0f89.pdf]

Evaluación del proyecto *Participación integral y ovitrampas en la prevención y control del vector de la Malaria y Dengue en Sayaxché*

Introducción 1
Conocimientos sobre dengue y malaria por parte de los pobladores 2
Participación comunitaria 4
Nivel de participación de líderes y vecinos, en algunos barrios 7
Labor del Programa de ETV´S 8
Actividades que realizan 8
Precepción de la labor del personal del Programa de Vectores 11
Cambios a partir de la capacitación recibida 12
Percepción sobre las ovillantas y trampas de control 12
Fortalezas y debilidades de la ovillanta 15
Problemáticas que presenta el personal de ETV´s para continuar con las ovillantas 16
Supuestos relevantes 18
Recomendaciones y conclusiones 20

Evaluación del proyecto "Participación integral y ovitrampas en la prevención y control del vector de la Malaria y Dengue en Sayaxché"

Introducción

El presente documento tiene como objetivo determinar la efectividad del control de dengue y paludismo, con el uso de ovillantas, trampas de control, así como la participación comunitaria en la localidad de Sayaxché, Petén. De igual forma, se muestra el nivel de aceptación por las herramientas de control, previamente mencionadas, y las problemáticas existentes durante su utilización.

La medición de tales objetivos se obtuvo a partir de técnicas de investigación cualitativa. Se seleccionaron ocho barrios, de los quince existentes. En cuatro se colocaron ovillantas y en los otros no. En los barrios con ovillantas se eligieron dos tipos de grupo, el primero —denominado "A"—, conformado con personas que tuvieron ovillantas en sus hogares; el segundo —"B"—, con individuos que no contaron con ésta ni con trampa de control. De igual manera, en los siguientes cuatro barrios se escogieron dos tipos de

agrupaciones: “C”, para habitantes que contaron con una trampa de control, y “D”, para quienes no obtuvieron ninguna de estas herramientas. En total fueron 16 grupos —4 “A”, 4”B”, 4 “C” y 4 “D”—.

A todos se les aplicaron grupos focales, se esperaba la presencia de doce personas en cada agrupación, pero la participación fue poca. En general, se les cuestionó respecto a sus conocimientos sobre estas enfermedades, su percepción sobre la participación, qué se hace en el barrio al respecto y el cambio en las visitas domiciliarias del personal de ETV´s. Además; al grupo “A” se le interrogó acerca de la percepción sobre el funcionamiento de la ovillanta y al “C”, sobre la trampa de control. Al “B” y al “D”, si habían sentido alguna disminución de la presencia de mosquitos *Aedes* y *Anopheles*, esto para conocer el impacto de la ovillanta y trampas de control en otros hogares.

Finalmente, se entrevistó al personal de ETV´s que colaboró en el proyecto, manteniendo enfoque en el cambio operativo en las visitas domiciliarias posterior a la capacitación, y la efectividad y/o las limitantes de las ovillantas colocadas en los domicilios.

Conocimientos sobre dengue y malaria por parte de los pobladores

El paludismo es una enfermedad que históricamente ha existido en el área de Petén —desde la existencia de la civilización Maya—; es por eso que en Sayaxché se tiene mayor conocimiento respecto a los síntomas y cómo tratarlo. Sobre dengue (o malaria) se sabe un poco menos, ya que se ha padecido en menor cantidad que la primera. Años atrás, se diagnosticaban muchos casos de paludismo y dengue —incluso varias personas murieron por lo mismo—, por lo que el personal del Programa de Enfermedades Transmitidas por Vectores (ETV´s) —conocidos como “los malaria”— llegaba a las casas a fumigar con DDT. Ahora se realiza en escasas ocasiones, esto genera molestia en la población, ya que lo consideran un método efectivo de prevención de enfermedades y, la mayoría, desconoce sus efectos negativos.

En general, el conocimiento que se tiene sobre el dengue y paludismo gira en torno a los síntomas, prevención con higiene en sitios y formas de tratarlo, ya sea con medicamentos o remedios naturales. Quienes proporcionaron mayor información al respecto, o trabajan en vectores o son colaboradores voluntarios (CV). En algunos casos se mencionó que éstas son la misma enfermedad, ya que los síntomas son parecidos. Además, desconocen qué tipo de

mosquito es el portador de tales padecimientos. La mayoría considera que cualquier mosquito puede contagiarlos. Asimismo, la minoría, al oír el término "malaria", automáticamente se refiere al personal de ETV´s. La conocen mejor como paludismo.

Para el paludismo, los síntomas que suelen reconocer son: fiebre, escalofríos, dolor de cabeza, vómitos, dolor de huesos. Para el dengue, fiebre, dolor de cabeza, alergia, vómitos, comezón, deshidratación, tifoidea, dolor de huesos. En el caso del hemorrágico, sangrado por nariz y boca, debilidad, falta de apetito. Las diferencias que registran entre una y otra son que en el dengue hay un tipo hemorrágico, que causa hasta la muerte y, mientras los síntomas del paludismo se presentan de manera cada dos días, con el dengue es constante.

Una cuestión relevante a mencionar es que las mujeres tienen mayor conocimiento al respecto; cabe recordar que ellas son quienes se encargan por velar la salud y bienestar de sus familiares. Según las prácticas culturales de la zona, los hombres se ocupan de trabajar y las mujeres del hogar, a pesar de que muchas también laboran profesionalmente. No obstante, señores que han padecido alguna de estas enfermedades, sí conocen al respecto.

Últimamente se han conocido diversos casos de Chikungunya —llamado por ellos como Chichinguya— en la población. Al ser un padecimiento del que se tiene poca información, ha generado mucha preocupación por sus habitantes. Además de que la consideran más grave que el dengue, incluyendo el hemorrágico. En todo momento externaron su interés por recibir información de la misma. Sobre todo cómo se transmite, ya que consideran que es por un mosquito diferente.

Tal desconocimiento respecto a estas enfermedades, y qué las transmite, afecta en la participación de la gente. Por un lado, al ver la constante permanencia del zancudo, sobre todo en las noches, consideran que la limpieza no es una estrategia relevante de prevención. Pese a ser un número reducido, hay quienes sostienen que el Aedes nace en charcos; por tal motivo, conciben de poca relevancia eliminar los recipientes que acumulan agua o limpiar las pilas y toneles.

Por otro lado, los diversos problemas existentes en el sector salud —como falta de medicamentos y mala atención proporcionada, ya sea por maltrato o mal diagnóstico[1]— han

[1] Uno de los entrevistados comentó que la atención que se recibe en las clínicas varía de acuerdo al nivel socioeconómico y las relaciones sociales que se tienen, siendo los de escasos recursos quienes mayor negligencia padecen. Como él mismo afirmó, "la sociedad es la sociedad y eso no lo vamos a cambiar…depende cómo es el sapo, así es la pedrada" (Hombre del Barrio el centro).

provocado desconfianza en la gente. Al ser la malaria y dengue enfermedades comunes en la zona y, dado lo anteriormente explicado, muchas personas se auto medican —ya sea por el conocimiento que tienen de estas enfermedades o bajo las recomendaciones que familiares o amistades les dan—. Cabe mencionar que existe un desconocimiento sobre la existencia de un establecimiento, en el tercer piso del mercado, para realizar la toma de muestra de gota gruesa y frote para la prueba de laboratorio (paludismo) de manera gratuita. Otros, prefieren acudir a las farmacias, contar al vendedor los síntomas que tienen y tomar el medicamento que ahí les sugieren. Sólo una persona mencionó acudir con el CV, quien es otra alternativa para realizar la prueba.

En general, desconocen los efectos negativos de auto medicarse, lo cual debe ser uno de los mensajes que se tendría que propagar. Respecto a los tratamientos utilizados, la mayoría refirió el medicamento; *acetaminofén* para el dengue y *paludol* y *primaquina* para el paludismo. En cuanto a remedios naturales mencionaron alka seltzer con limón, agua de coco, tres puntas (planta usada para combatir el paludismo), verbena, pimienta con canela, uña de gato (planta usada para combatir el dengue), bicarbonato con limón y miel, y agua de achiote. En general utilizan los remedios para ambas enfermedades. Cabe mencionar que, dado que varias personas no se diagnostican, en cuanto sienten la presencia de fiebre y dolor de cuerpo, toman el *acetaminofén*.

Respecto a la prevención, todos se enfocan en actividades de higiene en los hogares, como quemar la basura, dar vuelta a recipientes que acumulan agua, desechar las llantas y cáscaras de coco, el uso de pabellones, chapear los terrenos, podar los árboles, pintar la casa de blanco, lavar las pilas y toneles, el uso de "las trampitas" —ovillanta y trampa de control—, mantener limpio el hogar y usar abate en las pilas. No obstante, hubieron personas que manifestaron que nada se podía hacer para prevenirlo o que no servía la higiene si "por todos lados hay agua y el mosquito nunca se acabará". De igual forma, dos o tres entrevistados respondieron que una manera de prevención es tomar pastillas.

Participación comunitaria

Para la mayoría de la gente entrevistada, la participación es acudir a las reuniones y compartir ideas y conocimientos, ésta tiene mucha relación con la primera respuesta. Otra que se mencionó varias veces fue seguir las recomendaciones del personal de malaria y mantener

limpios los sitios. No obstante, pocos afirmaron que era solidaridad entre vecinos, hacer actividades que beneficien a todos o la convivencia. Esto habla de la poca percepción respecto a unir esfuerzos para el bien común (Ver Ilustración 1).

Ilustración 1 Definición de la palabra "participación"

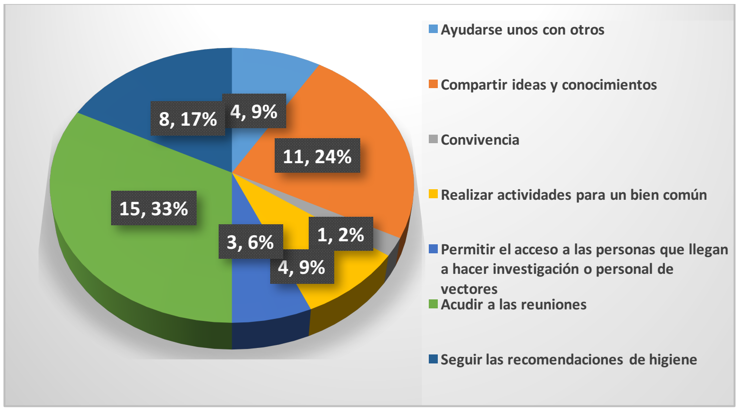

Fuente: Elaboración propia.

De forma recurrente, al preguntar qué consideraban por participación, la mayoría se quedaba callada y respondía brevemente. Durante el grupo focal en el barrio la pista, una persona —quien es parte del COCODE y de etnia maya— respondió lo siguiente:

> Tiene una palabra fundamental, la participación ciudadana. La palabra participación ciudadana es para participar en uniones, como dice el compañero, participar en trabajos, en hacer mejoras en el barrio, los sábados venimos para las faenas, también en nuestra religión participamos. Nosotros, como seres humanos tenemos nuestra religión, nuestra cultura, pero también el desarrollo. Entonces, no solamente es de hablar, porque teóricamente es de escuchar, pero también es llevarlo a la práctica. Entonces, la participación que estamos hoy acá contestando las preguntas y también apoyando a los compañeros. A la hora de venir a una reunión se aprende y el mensaje lo llevamos a la familia, los vecinos y ahí se hace la réplica. De ahí comunicamos, no solamente acá, sino también en la iglesia. Yo tengo un grupo de jóvenes y vemos cómo cuidar la salud, de cómo mantener el pueblo limpio. Yo creo que la participación es incluir a las mujeres, no ser el machito, hay que darles oportunidad que tengan el desarrollo. Hombres y mujeres participar y los niños que participen también. Todas actividades que se realizan que estén todos (Entrevistado en el barrio La pista).

Con lo anterior se puede apreciar cómo las condiciones sociales influyen en cada persona. Es muy dicho que en las aldeas, donde predominan los maya hablantes, existe mucha colaboración entre vecinos y todos asisten a las reuniones que fijan los líderes.

Estas prácticas son poco seguidas por los habitantes de Sayaxché, quienes, mayoritariamente, hablan castellano y sus prácticas culturales son diferentes o han cambiado.

Pese a no poseer mucho conocimiento respecto al concepto de participación, o no querer compartirlo, todos consideran que es importante para prevenir estas enfermedades. Afirman que sin la participación —enfocada a usar el pabellón y seguir las recomendaciones que les hace el personal de malaria— se seguirían enfermando. La mayoría refirió que asistir a las charlas es un medio relevante de aprender y transmitir la información a las familias de cómo evitar las estas afecciones.

Por su parte, la mayoría considera que "uno mismo" es responsable de evitar que el dengue y la malaria se sigan produciendo. En segundo lugar, se mencionó que era tarea de "los malaria". Por el contrario, muy pocos nombraron a las autoridades gubernamentales, quizás por la mala respuesta que han recibido de ellos. Son escasos quienes afirman que la prevención se trata de un trabajo en conjunto (Ver Ilustración 2). Generalmente se cree la preservación de la salud como una tarea que debe ser llevada a cabo de manera individual.

Cabe hacer mención de la importancia que el sector salud —refiriéndose al área de vectores— tiene en la comunidad, ya que fue muy mencionado, aunque también podría argumentarse que se ha creado una fuerte dependencia a las actividades que ellos realizan . Finalmente, quienes mencionaron a las autoridades de gobierno y al área de salud (a excepción de una mujer) fueron hombres. Siempre el enfoque de higiene y responsabilidad de prevenir enfermedades recae en el sexo femenino.

Ilustración 2 Responsabilidad en cuanto prevención de dengue y malaria

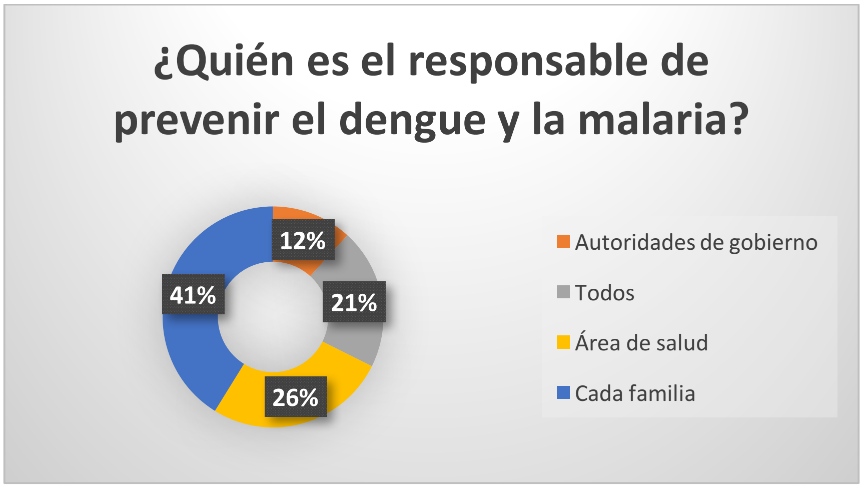

Fuente: Elaboración propia.

Gran parte de los entrevistados hicieron énfasis en la poca participación de sus vecinos al mantener sus terrenos y hogares sucios, considerándolos una amenaza a su salud. En teoría, podría afirmarse que se tiene una idea de prevención como un trabajo en conjunto. Pero en la práctica, difícilmente se organizan para realizar actividades de saneamiento o incluso no asisten a las reuniones que llega a hacer el COCODE. Muestra de la falta de participación es la poca presencia en los grupos focales realizados. Otra cuestión es que gente de barrios alejados del centro, y por ende con menor urbanización, se quejaron de la basura que habitantes del centro llega a tirar cerca de sus terrenos, como pañales y animales muertos. También se hizo mención de varios terrenos abandonados que no chapean.

Nivel de participación de líderes y vecinos, en algunos barrios

A continuación se muestra una tabla en la que se palma la percepción de los entrevistados respecto a la participación dentro de su barrio —ya sea en cuanto a higiene en el hogar y a las actividades que se realicen en el lugar—; la labor del COCODE y su preocupación por la higiene y salud[2].

[2] Se mencionan los 8 barrios donde se realizaron los grupos focales

Barrio	Higiene en el barrio	Participación de los vecinos	Percepción sobre la labor del COCODE	Preocupación en higiene y salud por parte del COCODE
El centro	50 % de los vecinos es higiénico	Poca	Casi no efectúa reuniones	No
	Mucha basura por las personas de otros barrios o comunidades que llegan todos los días, Sobre todo, les echan la culpa a los indígenas	No hay colaboración entre vecinos	Poco interés en su labor	
	Hay servicio de limpieza de calles, pero aun así se ensucia mucho			
El porvenir	Aproximadamente el 40% no mantiene la higiene en sus hogares, aunque ha mejorado	Hay personas que no dan apertura al personal de vectores	Poco interés en su labor	No
	Hay quienes tiran su basura en la calle, como pañales	Poca participación en actividades del barrio		El COCODE estaba ahí, comentó que sí
El triunfo	Últimamente ha habido mucha delincuencia	Muy poca participación	Mala	No
	Tiran mucha basura en la calle	Nada de unión, existen diversos grupos de vecinos		
	Poco aseo en las casas			
La pista	La mayoría mantiene limpios sus predios	Los maya hablantes son muy unidos	Buena	Sí
		Los que hablan castellano comentaron que los maya hablantes los hacen a un lado.	Los que hablan castellano argumentaron que no los convocan en sus reuniones	Quienes hablan castellano dijeron que no
El limón	Hay terrenos abandonados	No hay unión, comentaron que por la diferencia de razas (indígenas y	Mala	No
	Algunos vecinos no son limpios			
La unión	Se refirieron a los pañales que tiran en la calle	Poca participación	Poco interés en su labor	No
	La basura que les tiran los del centro	No hay unión, antes sí cuando eran pocos habitantes		
	Vecinos que no colaboran con la higiene			
Lomas del Norte	Vecinos que no son aseados	Buena participación	Casi no efectúa reuniones	No
	Terrenos abandonados	No hay unión entre vecinos	Las mismas señoras: que es bueno	Las mismas señoras: que habla de todo un poco
		Unas señoras afirmaron que sí hay unión y ayuda entre vecinos		
Santa Cruz	Gente que tiene terrenos abandonados	Poca participación	No conocen al COCODE, mala	No
	Del centro llegan a tirarles basura	Poca unión entre vecinos	No hace reuniones	
	La mayoría de vecinos cuida la higiene de sus predios			

Fuente: Elaboración propia

Respecto a los vecinos que no atienden las recomendaciones de higiene o que mantienen sus predios abandonados, los entrevistados afirmaron que es difícil llamarles la atención, ya que no quieren cargarse problemas con ellos. Desde la municipalidad no han recibido ayuda al respecto y, por ende, aseguran que el personal de malaria debería sermonearlos. Por otro lado, en general, la participación es mínima. Muchos aludieron a que varios habitantes asisten a las reuniones sólo cuando se regala algo, como pabellones.

Labor del Programa de ETV´S

Actividades que realizan

Como se mencionó previamente, la mayor parte de los habitantes en Sayaxché se quejan de la falta de fumigaciones. Anteriormente se hacía cada seis meses; ahora, sólo cuando se

diagnostica algún caso, esto con el fin de evitar el contagio de algún familiar o vecino. La comunidad se acostumbró a estas acciones; incluso consideran que con la fumigación se acabaría el problema de los zancudos y, por ende, estas enfermedades. Dada la crisis que se vive en el país, los pobladores creen que es la razón por la cual se ha disminuido esta práctica, denotando así el desconocimiento respecto a sus efectos dañinos en el medio ambiente.

> Porque esa fumigación es importantísima. Hace un tiempo hubo un zancudero que no se soportaba, porque nosotros decíamos “cómo es que no aparece malaria para echar humo”. Cómo beneficia, beneficia mucho, ayuda mucho. Porque el zancudo que se encuentra debajo de las mesas, ese sale, muere. Ya las mata. (Entrevistado en el barrio El porvenir).

Dentro de las actividades que realiza el personal que abatiza están: revisar la limpieza de los espacios—incluyendo pilas y toneles—, retirar los objetos que acumulan agua, colocar el abate en las pilas; a partir de este año, cambiar el agua en las trampas y las ovillantas —realizado sólo por un grupo de cuatro personas, quienes, además, tienen otras funciones—. Por su parte, la gente de promoción visita las escuelas y realiza actividades con los niños, como recolecta de basura en las calles —aproximadamente dos veces al año—; en las casas explican cómo prevenir las enfermedades —enfocado a limpieza de espacios—, qué tipos de mosquitos transmiten las enfermedades y cómo se reproducen.

La cantidad de visitas a hogares se realiza dependiendo del nivel de limpieza en el mismo. Las viviendas en donde se ha encontrado suciedad son visitadas cada semana. Por el contrario, los más limpios, cada 15 o 30 días. Regularmente en la época de lluvia, que hay más mosquitos, llegan con más frecuencia. Cabe hacer mención que, debido a problemas políticos y económicos en el país, el personal ha disminuido, lo que también ha provocado la baja en inspecciones. Por su parte, la revisión de ovillantas y trampas de control se ha realizado cada semana.

Si el hogar está limpio, los miembros reciben felicitación por parte del personal; en caso contrario, se les explica los riesgos de mantenerlo así y se les pide cambiar sus hábitos. Es importante señalar que, en diversas ocasiones, los abatizadores realizan las actividades que son responsabilidad de los habitantes, como limpiar las pilas, tirar el agua que tiene larvas, entre otras. Si bien, en momentos es una gran ayuda, sobre todo para los ancianos o

mujeres que no pueden cargar cosas pesadas, de alguna forma podría generarse una dependencia a ellos. Un ejemplo de esto se da con el manejo de la basura:

> Pero también los malaria tienen que ayudar, como hicieron la vez pasada que ellos iban a tirar la basura. Porque a veces nosotros no tenemos fondo de estar mandando la basura al basurero, porque hay que pagar Q30 mensuales, le cobran en la muni. Entonces si uno lo tiene, lo manda, si no, no. Entonces la vez pasada, ellos habían empezado a decir que por parte de los de salud iban a tirar la basura. Pero ahí está que una vez nos dejaron a los vecinos del porvenir, pasaron diciendo que juntáramos la basura. Sí a mí también. Yo saqué mi basura. Sí, y uno es obediente. Uno hace lo que nos dicen, pero ahí está que ellos no cumplen (Mujer, entrevistada en el barrio El porvenir).

Diversas personas argumentaron que existe un cambio mínimo o nulo respecto a la información que proporciona "malaria". Esto tiende a aburrir a los pobladores y a que no presten atención a lo que les comentan. "Es el mismo siempre [el mensaje]. Siempre dan las recomendaciones. Parecen discos rayados, lo que uno tiene que hacer, y ahí los escuchamos" (Mujer entrevistada en el barrio el limón). Esto podría ser una razón por la cual, pese a siempre estar asesorados por el personal, la gente desconoce mucho sobre estas enfermedades. Aunque algunos refirieron escuchar mensajes radiales o haber visto algún cartel sobre prevención, la mayoría argumenta que sólo con las visitas domiciliarias obtienen conocimiento.

De igual forma, la gente considera que las actividades de abatización han sido las mismas desde años atrás. No obstante, reconocen cambios que se han ido implementando por el bien de la población, como la entrega de pabellones, introducción de peces en las pilas —aunque no ha funcionado por la cloración del agua—y, ahora, las trampas de control y ovillantas que se colocaron en algunos hogares.

Si bien el personal de ETV´S se encarga de revisar la higiene en los hogares y emitir recomendaciones, no tienen la autoridad de obligar a la gente seguir las indicaciones. Sin embargo, en diversas ocasiones, entrevistados se quejaron de que estos no hacen algo con los vecinos que no son higiénicos. Esto es parte de la dependencia que se ha generado con el Programa y de la poca respuesta que se tiene por parte de la municipalidad.

> Yo he visto que si van enfrente de mi casa, ese don ni lava la pila ni el tonel, la pila verde. El baño está por encima, donde van a hacer sus necesidades…Yo ya he hablado [personal de malaria] con ellos, que por favor vayan a hablar con él, que haga otro baño

> o cómo hace. Ellos no hacen caso…Como dice mi mamá, es que los de la malaria tienen que ver eso, la salud pública tienen que ver eso (Mujer entrevistada en el barrio Lomas del Norte).

Precepción de la labor del personal del Programa de Vectores

En general, el Programa de Vectores tiene muy buena reputación ante la localidad. La gente está consciente de que gracias a su trabajo, los casos de dengue y malaria se han reducido notoriamente. A continuación algunos comentarios de los entrevistados:

> "…ha ayudado mucho la cuestión educativa que nos ha dado malaria, cómo evitar que se propague más la enfermedad. Malaria ha ayudado mucho, tienen una forma de trabajar casi militar, lo que sí ya no fumigan" (Entrevistado en el barrio El centro). "Muy buena, muy agradecido con malaria" (Entrevistado en el barrio el porvenir). "Ahora casi ya no da dengue, pero muchos años atrás esto fue muy popular. Pero gracias a Dios y al centro de salud y las personas que han estado apoyando, esto se ha venido mejorando. Los casos ya son salteados, con la ayuda de ustedes" (Entrevistada en el barrio el limón). "Bien. Sí, porque es la única institución que ve por la salud de la gente. Porque si no fuera por ellos, nosotros estaríamos llenos de malaria" (Entrevistado en el barrio El porvenir). "Yo creo que es buena, porque ahí también nos ayudan. Nos han dado pabellones, nos explicaron cómo usarlo, pero hay gente que ni siquiera los pone y los zancudos siguen" (Entrevistada en el barrio El limón).

Como en todo, no existe la perfección, algunos consideran que sería mejor que explicarán más a las familias; podría apreciarse que las actividades de abatización y promoción se han realizado, por algunos, de manera mecanizada, lo cual tiende hacer perder el interés de la gente. Hace falta mayor información sobre prevención; no sólo enfocada a la limpieza del sitio, sino a la higiene personal y el trabajo comunitario. Sobre todo, socializar más el proyecto de las ovillantas. Para que más personas conozcan su función. Por otro lado, hubo quejas respecto al vaciado de agua:

> Es que uno hay que pararse también, ellos son bien abusivos, hay que tratarles mal. A veces tengo mi agua de lluvia, porque nosotros somos pobres, no tenemos para comprar agua, y llegó un abusivo de ellos, no me preguntó y me tiró el agua. Pero lástima que no lo miré, porque uno en su casa manda, usted es la dueña de su hogar y vengo yo y boto sus cosas abusivamente, usted se enoja (Mujer entrevistada, barrio El porvenir).

La falta de agua es un problema que afecta a toda la comunidad; vaciar el agua con frecuencia, obviamente genera molestia en la gente. Sería idóneo buscar otras alternativas, como colarla o reciclarla. De igual forma, se comentó el hecho de que algunos trabajadores del programa

no son aseados en sus hogares, por lo cual pueden desacreditarse ante la población. Ellos deberían ser un ejemplo de buenas prácticas de prevención.

Cambios a partir de la capacitación recibida

Frente al taller recibido, el personal de ETV´S dice estar muy agradecido y motivado. Argumentan haber recibido muchos conocimientos que aplicarán. No obstante, hasta el momento no se ha visto alguno. Sin embargo, quienes se encargaron del cuidado de las ovillantas y trampas de control, buscaron el involucramiento de la gente, lo que dio muy buen resultado. Además, les explicaron que esto es una herramienta para evitar enfermedades, pero que deben continuar con las labores de higiene en los hogares, para su buen funcionamiento.

Finalmente, el personal se enfocó en el mantenimiento de la ovillanta y dejó a un lado la mejora de la participación comunitaria y el involucramiento de las autoridades del barrio. Últimamente comenzaron a incluir a los miembros de la familia, pero aún hace falta más trabajo al respecto. Sobre todo con quienes aún desconocen el fin de esta investigación. Cabe resaltar la falta de personal para poder completar estas actividades y la falta de acompañamiento por parte de miembros del proyecto.

Percepción sobre las ovillantas y trampas de control

Todas las personas entrevistadas, que tuvieron ovillanta y/o trampa de control, se mostraron enormemente satisfechas y recomendarían su uso a otros vecinos. Incluso, muchos manifestaron interés por saber la cantidad de huevecillos encontrados cada semana. Como se hizo mención anteriormente, regularmente las mujeres se involucran más en las actividades de salud. En el caso del manejo de la ovillanta, se contó con la participación de varios hombres y niños. Les interesó conocer más sobre su uso y cómo mantenerla en buen funcionamiento. En ocasiones, cuando el personal llegaba a hacer el cambio de agua y papeleta, las personas ya lo habían realizado. El hecho de haber encontrado huevecillos generó en ellos mayor interés. Aunado a lo anterior, el miedo por contraer Chikungunya y Zika ha fomentado mayor involucramiento.

Al principio, por curiosidad, los niños tiraban el agua de las ovillantas. Una vez que sus padres hablaban con ellos y les explicaban su funcionamiento, dejaban de hacerlo. De igual forma, las personas transmitieron la información a sus familiares y amigos cercanos. A pesar de eso, aún son muchos quienes las desconocen.

> Hubo una ocasión donde sale el señor, que habían tirado el agua a la trampita y la habían puesto en la esquina, pero fue el suegro de él que no sabía para qué está la trampita. Y el suegro dijo que los de malaria andan diciendo que desechen recipientes que acumulen agua y por eso quitó la trampita. Él mismo le llamó la atención, pero antes le explicó para qué era y lo volvió a poner. A raíz de que el señor ya tenía el mensaje, le pudo explicar para qué era (Trabajador del Programa de ETV´s).

Vecinos que pudieron conocerlas empezaron a pedirlas, el problema fue que sólo se colocó un número específico. Sin embargo, se les informó que tal herramienta influía en su bienestar al contrarrestar el número de mosquitos en la zona. Al parecer, abarca sólo un área pequeña; no tiene mucha cobertura.

> Sí, de hecho cuando voy a abatizar, hay quienes me dicen "¿por qué doña fulana tiene de esas trampitas y yo no?". Ya viene uno y les explica. "Pero también a ustedes les beneficia, no sólo a esa familia". Ahí les explica uno. Sí se les dice que llegará un momento en que se les tomará en cuenta. Me dicen, "si hubiera oportunidad, tómenme en cuenta para tener una trampita". Sí se ve la aceptación de la gente. Incluso los abatizadores nos han comentado que a ellos les preguntan por las trampitas (Trabajador del Programa de ETV´s).

Puesto que tales insumos fueron colocados en pocos hogares y no se convocó a una reunión para dar a conocer el proyecto, a excepción de quienes los tuvieron y vecinos y/o familiares cercanos, nadie más las había visto o escuchado. Después de explicarles el funcionamiento y la buena aceptación que había tenido, los entrevistados expresaron su interés por tenerlos en sus hogares. Siempre y cuando el personal de vectores les suministre el material necesario —agua, papeletas, atrayente—.

Una vez conocido la aceptación que las ovillantas y trampas de control tuvieron en los pobladores entrevistados, se les comentó la dificultad que tendría el personal de vectores en visitar los hogares para realizarle el mantenimiento requerido, como limpieza, cambio de agua y papeleta. Asimismo se les explicó que antes de proporcionarles el producto, en caso de que el proyecto logre prolongarse, se realizarían reuniones de capacitación para su uso y conservación. Seguidamente, se les preguntó la posibilidad de contar con su participación en estas actividades. Al respecto, la mayoría respondió que, con previa capacitación y provisión de insumos, estarían dispuestos a colaborar. Sólo unas personas de los barrios El centro y El

porvenir (una mujer y cuatro hombres) dijeron que no lo harían. Sus razones fueron falta de tiempo o no querer cargarse un compromiso así.

En cuanto a la participación de los demás vecinos —que no asistieron a la reunión o no fueron requeridos—, argumentaron que, al ser por el bien de su salud, estarían dispuestos a participar, aunque siempre existirán quienes de plano no lo hagan. Es menester señalar que los habitantes de Sayaxché, al menos la mayoría, están acostumbrados a que se les proporcionen las cosas[3]; es por eso que, si se convocara a una reunión de socialización del proyecto, al saber que se les obsequiarían las ovillantas, la participación sería total. El hecho de darles un insumo más de prevención, podría ser motivador para que generarán un interés por colaborar en su mantenimiento.

	Barrio	Percepción de funcionamiento	Factores externos que influyen
Con ovillantas	El centro	Se encontraron muchos huevecillos. Percepción de misma cantidad de zancudos	Negativamente: suciedad en las calles
	El porvenir	Se encontraron muchos huevecillos. Percepción de misma cantidad de mosquitos	Positivamente: colocar la ovillanta en lugares estratégicos
	El triunfo	Se encontraron muchos huevecillos. Percepción de la misma cantidad de mosquitos	Negativamente: las pozas donde se acumula agua, existencia de muchos negocios, cercanía con el río, época de lluvia
	La pista	Se encontró menor cantidad de huevecillos. Percepción de disminución de mosquitos	Positivamente: limpieza del sitio. Negativamente: agua encharcada en las calles, falta de drenaje
Con trampas de control	El limón	Se encontraron pocos huevecillos. Percepción de misma cantidad de zancudos	Negativamente: algunas personas no tienen sanitarios, época de lluvia
	La unión	Se encontraron pocos o cero huevecillos. Percepción de la misma cantidad de mosquitos	Negativamente: basura que llegan a tirar, desplazamiento de personas a otros barrios o localidades, época de lluvia, terrenos abandonados
	Lomas del Norte	Se encontraron muchos huevecillos. Percepción de disminución de mosquitos	Positivamente: higiene en la casa. Negativamente: agua encharcada en las calles
	Santa Cruz	Se encontraron pocos huevecillos. Percepción de misma cantidad de mosquitos	Negativamente: desplazamiento de personas a otros barrios o localidades, basura que llegan a tirar

[3] Sayaxché fue uno de los poblados afectados durante la guerrilla en Guatemala. Una vez que se le dio fin, sus pobladores recibieron ayuda de diversas ONG´s, con el fin de mejorar su calidad de vida. Esto generó en ellos un apego a las prácticas paternalistas, a grado tal de negarse a hacer las cosas, que les generará beneficios, por sus propios medios.

Pese a que no todos los entrevistados lo mencionaron, los elementos que influyen en el funcionamiento de la ovillanta son: la cercanía al río; después de la época de lluvia, cuando el agua baja, incrementa el número de zancudos, al igual que durante la noche. La migración interna e internacional, incluso las inter barriales, son causales de la prevalencia de dengue y malaria en la zona. Por su parte, la falta de letrinas, en algunas casas, también afecta negativamente[4], además del encharcamiento de agua en algunas calles, debido a la falta de urbanización.

Todos los entrevistados se quejaron de la prevalencia de mosquitos; son pocos quienes argumentaron haber percibido una disminución. Incluso, algunos comentaron que su creencia era que con la instalación de la ovillanta se acabarían los mosquitos. Sería prudente tomar en cuenta esta cuestión y dar a conocer que no sucederá, ya que de lo contrario, las personas podrían generar falsas expectativas y considerar improductivo el cuidado de la ovillanta.

Fortalezas y debilidades de la ovillanta

A continuación se presentan, en general, las fortalezas y debilidades que encuentran, tanto el personal de ETV´s como quienes la tuvieron en su hogar.

	Fortalezas	Debilidades
**Percibidas por la población**	*Instrumento para combatir la enfermedad. Es una forma de cuidar nuestra salud.<br>*El zancudo ha mermado bastante. Creemos que por esa razón.<br>*El mensaje que da para poder realizarlo con cosas que uno tiene en casa. Cualquiera puede tener una llanta, son cosas reutilizables.<br>*Le ayudaría para destruir los huevecillos del zancudo.<br>*La conciencia que genera en un mismo para la prevención.	*Se debe cuidar que el perro o los niños no lo agarren<br>*Luego llegan los de malaria a revisarlas y uno no está en su casa. Entonces sería que cada uno sea el supervisor. Pero, hay gente que no tiene ese interés y responsabilidad para revisar eso. Ahí es de cada uno.<br>*Cuando atrapa los huevecillos de los zancudos, no atrapa los otros zancudos. Sería bueno para atrapar otros zancudos.

[4] Si bien algunos COOCDES han gestionado apoyo para esto, los habitantes tienen que dar la mitad del presupuesto y se niegan a hacerlo. Otros, por prácticas culturales, se resisten a su uso.

**Percibidas por el personal de ETV´s**	*Menos personas enfermas, se va bajando la población del zancudo que transmite las enfermedades<br>*Participación y aceptación de las trampitas	*Los niños tiraban el agua<br>*Muchos animales llegan ahí a tomar agua<br>*No atrae *Anopheles* ni *Culex*

Fuente: Elaboración propia.

Problemáticas que presenta el personal de ETV´s para continuar con las ovillantas

Los problemas que el personal de ETV´s tuvo para la ejecución de su trabajo, principalmente, fueron la falta de recursos —como vehículos y gasolina para desplazarse, suministro de agua— y de personal. En época de lluvia se dificultan las cosas, ya que hay zonas en donde el estado de las calles, generalmente de terracería, dificulta su traslado. Aparte del mantenimiento de las ovillantas, ellos deben cumplir otras actividades, como abatización, promoción, conteo y captura de casos. Esto les genera algunas complicaciones; tienen que realizar sus actividades rápidamente para poder lograr los objetivos. Sólo tienen tres días para revisar las ovillantas —los otros dos días son usados para llenar las boletas—, pero algunas veces han encontrado viviendas donde deben explicar a sus visitantes el uso de la ovillanta; eso ocasiona mayor retraso.

En otro aspecto, debido a la alcalinidad del agua y a que luego está muy clorada, sólo se puede usar de pozo —o lluvia—, pero no todos poseen uno en su hogar. Por tal motivo, conseguir agua de pozo genera dificultades. Sobre todo si se piensa incrementar el número de hogares con ovillantas. Un empleado del Programa de Vectores apoyó con el insumo, lo cual le suscitó costos en electricidad, al utilizar una bomba. Resulta necesario buscar alternativas ante esta problemática.

Aunque en general no tuvieron inconvenientes con la aceptación de la gente, el personal de vectores señaló que en pocas casas la gente se aburrió con la ovillanta y decidieron colocarla en otro hogar. De igual forma, se cambiaron de sitio en casas donde hubo inquilinos que se mudaron. Otra cuestión fue que, llegar todas las semanas y entrar al hogar para revisar las ovitrampas y ovillantas, incomodó un poco a los habitantes, al igual que al personal. No obstante, buscaron otra forma de entrar a los patios sin invadir la privacidad de la familia o, de plano tuvieron que cambiarlas.

Por otro lado, el atrayente colocado en las ovillantas debe ser enviado desde México, lo cual, en ocasiones, les ha generado un costo extra al tener que pagar un impuesto en la

frontera. Les preocupa, en caso de que incremente el número de ovillantas en los hogares, de qué manera abastecer con atrayente a toda la población. Consideran que es una actividad que los habitantes no podrían hacer; por ende, debe haber personal encargado de eso.

A continuación se presentan las problemáticas externas que afectaron el uso de la ovillantas y los defectos encontrados en éstas.

Factores externos que influyeron en su uso	Problemáticas encontradas en la ovillanta y sus componentes
Se meten arañas, cuijas o ranas; así no llegan los mosquitos.	Al principio se echaba 4ml de atrayente, lo que generaba una lama y luego se llenaba de gusanos.
Por el olor del atrayente, al principio, unas ratas hicieron sus nidos ahí	El atrayente de *Anopheles* no funcionó
El calor hace que la cantidad de agua en la ovillanta disminuya	Se entregaron ovillantas de diferentes tamaños, les funcionó mejor el de más grande
En época de lluvia aumenta el número de mosquitos; al parecer, la ovillanta no puede controlarlo	Algunas ovillantas defectuosas, presentaron filtración de agua o estaban muy aguadas (descorchadas), se torcían
	El pegamento usado en el tubo donde pasa el agua no funcionó. Tuvieron que usar jabón para evitar la filtración.
	El papel pellón no se encuentra en la zona. Por ahora se estará probando con toallas de papel para cocina
	El tamaño del papel pellón tuvo que disminuirse

Fuente: Elaboración propia

Aparte de valorar las problemáticas surgidas durante el tiempo que se han mantenido las ovillantas, el personal de ETV´s sugiere que es importante valorar los inconvenientes económicos, y por ende, de abastecimiento de material que existen en el programa. Incluso se les dificultó conseguir hojas para imprimir las cartas de invitación a los grupos focales. Por tal motivo, sería prudente tomar en cuenta un rubro de apoyo económico para material.

Cabe hacer mención que, pese a tales obstáculos, los empleados buscaron los medios para realizar sus actividades. Existe mucha voluntad de obtener buenos resultados con este proyecto.

Por último, las personas que contaron con una ovillanta, sugieren de gran importancia que se "socialice el proyecto", convocando a todos los habitantes del pueblo a una charla informativa al respecto. Aún se desconoce el uso de esta herramienta de prevención de dengue y malaria.

Supuestos relevantes

1. **La higiene en la vivienda no beneficia** porque los moquitos se crían afuera en lugares naturales.
2. **El enfoque de higiene y responsabilidad en la prevención recae en la mujer.**
3. **Baja aceptación y satisfacción de los servicios de atención médica** ministerial. Preferencia por la **automedicación** como alternativa inmediata. "Depende del sapo es la pedrada": La atención depende del nivel socioeconómico y de las relaciones sociales.
4. **La falta de información sobre un problema** genera preocupación y actitud por conocer y participar. Como también, buscar alternativas disponibles (automedicación, remedios, etc.). Generar mayor información sobre prevención; no sólo enfocada a la limpieza del sitio, sino a la higiene personal y el trabajo comunitario.
5. **Dependencia del control por el Programa-"Palúdicos"** (vertical), sin participación. Consideran las fumigaciones una alternativa para acabar con el zancudo y la enfermedad. Los "abatizadores" realizan las actividades que es responsabilidad de los habitantes. Desconocimiento o baja percepción de la utilidad o beneficio del CV y el sitio para la toma de muestra de paludismo en el mercado principal (centro) de Sayaxche.
   No tienen autoridad para regular la responsabilidad de los habitantes sobre medidas de higiene y prevención, que explica, en parte, esta dependencia que se ha generado con el Programa y de la poca respuesta que se tiene por parte de la municipalidad.
6. Baja percepción de ventajas sobre el **trabajo comunitario y solidario**. Origina acciones insolidarias (tirar basura en el terreno del vecino u otro barrio, terrenos abandonados, falta de limpieza, orden y chapeo de patios y terrenos, tirar la basura en la calle, etc.) e individualistas "Los indígenas son los sucios". "Los mayas son muy unidos", "asisten a reuniones cuando les regalan algo, como los pabellones"
7. **Conocimiento de medicina tradicional** (uña de gato-dengue, etc.).
8. **Comunicación e interacción de acuerdo al perfil étnico-cultural** (lenguaje y costumbres). "la información que proporciona "malaria", tiende a aburrir a los pobladores y a que no presten atención a lo que les comentan…es el mismo mensaje

de siempre….parece disco rayado.” “la mayoría argumenta que sólo con las visitas domiciliarias obtienen conocimiento”.

9. **Alternativas aceptables.** “reconocen cambios que se han ido implementando por el bien de la población, como la entrega de pabellones, introducción de peces en las pilas —aunque no ha funcionado por la cloración del agua—y, ahora, las trampas de control y ovillantas que se colocaron en algunos hogares”.
10. **Estrategia de control con Ovillantas.**
    a. Comunidad.
        i. Falta de agua es un problema en la comunidad, vaciar con frecuencia genera molestia en la población, sería adecuado buscar alternativas como el reciclaje del agua.
        ii. Satisfacción por la ovillantas y ovitrampas; recomiendan su utilidad a otros vecinos.
        iii. Interés por conocer la cantidad de huevecillos recolectados cada semana.
        iv. Las mujeres involucradas en actividades de salud.
        v. Interés por conocer su uso y mantenimiento.
        vi. Quieren poseer las ovillantas en sus hogares. Siempre y cuando el personal de vectores les suministre el material necesario.
        vii. Implementación y mantenimiento por la familia. La mayoría respondió que, con previa capacitación y provisión de insumos, estarían dispuestos a colaborar o participar. Sólo unas personas de los barrios El centro y El porvenir (una mujer y cuatro hombres) dijeron que no lo harían. Sus razones fueron falta de tiempo o no querer cargarse un compromiso así.
        viii. El hecho de darles un insumo más de prevención, podría ser motivador para que generarán un interés por colaborar en su mantenimiento.
        ix. Percepción de efectividad. Muchos huevecillos en ovillantas y pocos en ovitrampas.
        x. Funcionamiento relacionado con cercanía al río, después de la época de lluvia, cuando el agua baja se incrementa el número de mosquitos, durante la noche.
        xi. La movilidad y migración interna e internacional influyen en la prevalencia del dengue y malaria. La falta de letrinas, encharcamiento en calles, falta de urbanización influyen en el problema.
        xii. Creencia que la instalación de ovillantes acabaría con los mosquitos.
        xiii. Socializar el proyecto con la comunidad y autoridades de Sayaxche
    b. Programa ETv´s.
        i. La capacitación en Ecosalud en ETV´s motiva y fortalece el trabajo en campo, mejorando el involucramiento de la población con las ovillantas y manteniendo la participación en la prevención, incluyendo a las autoridades municipales.

ii. Escaso recurso financiero, vehículo, combustible y personal. Mayor dificultad durante la época de lluvia
iii. Mayor de acompañamiento de investigadores en campo.
iv. Adecuar la herramienta estratégica para su implementación sostenida, en particular sobre atrayentes (disponibilidad, funcionamiento y costos), papeletas o papel pellón (disponibilidad y costos) y herramienta (filtración, pegamientos, etc.) y formatos de registro.

Recomendaciones y conclusiones

- En general, la información que tiene la gente acerca del dengue y malaria es escasa y, en algunos casos, errónea. Se deben realizar estrategias para tener la atención de la población, las recomendaciones típicas que efectúa el personal de vectores tiende a aburrirlos. Una manera podría ser crear una página en Facebook, la mayoría posee una cuenta y celular con internet, donde se publiquen recomendaciones pequeñas y se contesten dudas. También, se tendría que generar alianzas con líderes religiosos y organizar jornadas de limpiezas, así como reuniones informativas.
- Para evitar que se auto mediquen, es importante dar a conocer el establecimiento que está en el mercado, así como promover la importancia del CV. Aunado a esto, sería relevante involucrar más al CV en actividades realizadas con la población y el personal de ETV´S. Para que tenga mayor presencia.
- Pese a tener muy buena reputación, es relevante la imagen que el personal de ETV´s da a la población. Por lo cual, deben dar el ejemplo con prácticas de higiene. Además, sería idóneo que buscaran alternativas para la reutilización del agua.
- La participación de la población es un tema que se debe trabajar mucho. Sobre todo el individualismo y conflictos por etnias. Por otro lado, al momento de generar actividades con la población, es relevante tener presente las diferencias existentes en cada barrio. De igual forma, tomar en cuenta a los maya hablantes; contar con un traductor en las reuniones y elaborar folletos informativos en maya.
- Generar estrategias para reducir el número de terrenos abandonados y vecinos que no son higiénicos. Aquí es muy importante la colaboración del COCODE y el ayudante municipal. Se podría establecer sanciones a quienes no cumplan con las indicaciones —elaborar un reglamento de prácticas de higiene comunal—; por ejemplo, podrían gestionar su regulación municipal con notificaciones, multas hasta, en casos

extremos, la confiscación de los terrenos no cuidados y darles un uso que traiga beneficios al barrio. Rentar el terreno y usar el dinero para la recolección y reciclaje de basura en los barrios. Sería muy bueno dejar en claro que el ayudante municipal no tendría autoridad sobre el terreno y el dinero obtenido, sería para beneficio de la comunidad. Los COCODES deben tener mayor abordaje en temas de salud y mejorar su imagen y labor frente a sus vecinos.

- Tratar de cambiar las prácticas paternalistas, sobre todo las que ejecuta el personal de vectores. Aplicando los principios y beneficios del enfoque ecosistémico.
- Importante, establecer mecanismos para que la población no tire basura en la calle y empiece a reciclarla. Esto se podría empezar con los niños en las escuelas. Enseñarles manualidades con objetos reciclados y realizar más jornadas de recolección de basura en los barrios.
- Tomar en cuenta las problemáticas encontradas en las ovillantas, para mejorar su uso.
- Para poder continuar el proyecto, es necesario tomar en cuenta los problemas económicos y de personal que existen en el Programa de Vectores.
- Socializar el proyecto, enfatizando los objetivos a cumplir y la participación de la gente para lograrlo. Hacerles ver que con esto, no se eliminarán todos los mosquitos.
- Es importante colocar más ovillantas en cada barrio, ya que éstas no tienen mucha cobertura.
