# Supplementary material for: Preliminary results on the control of Aedes spp. in a remote Guatemalan community vulnerable to dengue, chikungunya and Zika virus: community participation and use of low-cost ecological ovillantas for mosquito control
Source: F1000Res. 2017 Feb 22;5:598. Originally published 2016 Apr 7. [Version 3] doi: 10.12688/f1000research.8461.3 (PMC5225411; doi:10.12688/f1000research.8461.3)
Supplement: Peer review history

# Peer review: Referee response for version 2

**DOI:** 10.5256/f1000research.11232.r18573

**Published:** 2017-01-10

**Authors:** Leon Eklund Hugo

**Affiliations:** 1 Mosquito Control Laboratory, QIMR Berghofer Medical Research Institute, Brisbane, Qld, Australia

I acknowledge the authors' change to the title to reflect that the study is ongoing and the results are preliminary, the publication is certainly a preliminary report. The other reviewers raise some legitimate concerns over the study design and statistical treatment of the data. I felt more could be done in the current version to at least address some of the legitimate concerns over the choice of statistical tests used (e.g. t test and linear regression analysis) and urge the authors to address these concerns to correctly present and comment on the preliminary data they currently have on hand. I also recommend the graphs are improved to attain publication quality (suggest using publication quality graphing software) and that the authors clearly label x and y axes. I struggled to follow when the data represented total counts for all traps combined or were for individual units, for example. I strongly suggest that the experimental design concerns raised by the reviewers are being addressed in the ongoing study so that the authors can confidently measure the trapping improvements made by the ovillantas.

I have read this submission. I believe that I have an appropriate level of expertise to confirm that it is of an acceptable scientific standard, however I have significant reservations, as outlined above.

# Response

**Published:** 2017-01-27

**Authors:** Gerard Ulibarri

**Affiliations:** Laurentian University, Canada

Let us not forget that the primary objective was the study of the acceptance, by the community, of the methodology used to collect mosquito eggs, in order to obtain a good and sustainable surveillance and vector reduction.  Thus the original design of the study was based on the community acceptance **and participation** of the intervention solely.

 The creation and use of the ovillantas was only a secondary necessity, due to the last minute withdrawal of support from a Canadian company, with the originally planned modified ovitraps (which had already been tested in Canada and Mexico, data being prepared for publication).

 To our surprise, the ovillantas were well accepted by the community, thus the study was labeled a success, according to our initial experimental design.  Nevertheless, we recognize that more work needs to be done in the field of community participation.

 The use of the ovillantas provided some puzzling results, collecting large amounts of *Aedes* eggs, thus we decided to rapidly run a simple test on the null hypothesis of the intervention as a whole. 

 We used the independent simple t-test simply to find out if the average between the control site and the intervention site was significant and statistically meaningful in the short 10 months duration.  This simple test has provided us with a positive answer that will allow us to continue expanding, with confidence, on the intervention’s approach to community participation/personnel training and modified ovitrap use.

 In future studies, with more data and a better designed study on the use of the ovillantas, we plan to use other statistical analysis, such as two-way ANOVA, Mann-Whitney test or another statistical test that allows to corroborate the null hypothesis that the ovillantas are more attractive than the standard ovitrap.

 We do not consider necessary to describe these paradigms in the paper, given that only preliminary results are shown.  It must be clear to the reviewers that we have not claimed that the ovillantas alone are much better than the standard ovitrap.  More data and testing are required to achieve this goal, and this idea is included in our future plans.

 We feel that it is not necessary to expand on explaining, at this point, our choice on the use of a simple test to find the statistical significance given the quantity of data recollected so far.  We hope this will suffice to the Reviewers to accept that we are still working on obtaining the required data, and that the objective of this preliminary publication has been the sharing of positive results using a tool affordable to everybody, in the midst of a crucial viral dissemination by mosquitoes.

 Figures 2 and 3 have been modified and the authors hope this is enough to proceed with the publication, thank you.

# Peer review: Referee response for version 2

**DOI:** 10.5256/f1000research.11232.r18928

**Published:** 2017-01-04

**Authors:** Anna M Stewart Ibarra

**Affiliations:** 1 Center for Global Health and Translational Science and Department of Medicine, State University of New York Upstate Medical University, Syracuse, NY, USA

The authors have made sufficient revisions to merit Approval of the manuscript.

I have read this submission. I believe that I have an appropriate level of expertise to confirm that it is of an acceptable scientific standard.

# Peer review: Referee response for version 1

**DOI:** 10.5256/f1000research.9111.r16060

**Published:** 2016-09-02

**Authors:** Johannes Sommerfield

**Affiliations:** 1 Special Programme for Research and Training in Tropical Diseases (TDR), World Health Organization, Geneva, Switzerland

This article describes an intervention carried out in rural Guatemala consisting of comparing standard vector control against an integrated delivery of a) web-based health worker training, b) low-cost ovitraps (“ovillantas”) and c) community engagement. I agree with the other reviews and add the following:

 The title is quite long. In the introduction make clearer that the modified ovitraps are called “ovillantes” (the photo shows that but not in the introduction). To what extent do the authors think the ovillantes can be replicated in other settings, e.g., urban? Why did the authors choose to work on *Aedes aegypti* in a rural area, although *Aedes aegypti* is mainly an urban vector? Would ovillantes be applicable in cities, in Guatemala or elsewhere in Latin America?

 Pertinent research project in times of arboviral disease epidemics, however, under methods more clearly describe study design and indicators used to measure intervention effectiveness and detail methods, including methods in qualitative research. There is now growing evidence that community interventions can in fact have an impact on the disease incidence and serological parameters  (recent publication on Nicaragua), so there is a need for good research and strong evidence on the effect of community interventions. This project did not do that, clearly spelled out in the discussion section, but the methodology section could be strengthened by explaining the analytical (and not only descriptive) elements of the study design.

 The results suggest that ovillantes are more effective than standard ovitraps by comparing both interventions in different neighborhoods (“study sites”). Clarify whether this is by cluster-randomization or not.

 Focus groups results are presented to show (positive) impact on social participation but they are not presented by intervention areas so that the effect of the intervention on social participation  cannot clearly be attributed.

 As the article argues, the FGD results “can help guide future strategies geared to strengthen community participation” but they do not fully indicate an  impact of the intervention. Also, while referring to the “community” and social participation the article refers to different ethnic groups and certain “communication difficulties”: unclear what this refers to (aren’t all of the communities of Mayan descent? What sub-groups or other ethnic groups is the article referring to?).

I have read this submission. I believe that I have an appropriate level of expertise to confirm that it is of an acceptable scientific standard, however I have significant reservations, as outlined above.

# Response

**Published:** 2016-11-30

**Authors:** Gerard Ulibarri

**Affiliations:** Laurentian University, Canada

Comments from the authors are accommodated following the reviewers 'paragraphs'

 Title has been modified.

 Sayaxche is a small city (urban) in an isolated area of Guatemala (rural).  All study was carried out within the city limits.  Sayaxche was chosen because the high dengue cases in previous years. 

 Yes, there are plans to implement the ovillantas in other cities, in Latin America, Asia, South Pacific, etc.  Wherever the *Aedes* mosquitoes are a problem, some people are already doing so.

 ​​​​​​This is a publication explaining the preliminary results from a three-prong study, a new publication will deal with the technical parts of the study, when data is more abundant.

 We know that this publication might have been premature, when dealing with pure scientific data. We trust that publishing these preliminary results early, could help more people given the urgency. We believe that together we could learn more about the implementation of this simple/affordable way to reduce *Aedes* population, and in turn provide tools aimed at protecting vulnerable people

 ​​​​​​Given the size of the city of Sayaxche, the ovillantas setting was ‘randomized’ in a manner that most of the city was covered. Although, a slight preference was given to sections of the city where most affected by dengue, based on previous years local data.

 The aim of the study was to ‘study’ social participation at the same time as the effect of the ‘new’ ovillantas on amount of mosquitoes produced in the area. The social participation was clearly present, the effect of the ovillantas was clearly demonstrated.  Now, we need to study the long term effect of the combined methodology.

 ​​​​​​In this study in particular, we encountered a cultural ‘division’ among the different members of the community.  At times, we required a translator to communicate with the ‘participants’ of the study, given that their mother language was Mayan, not Spanish.  This difficulty might  or might no be important, but needs to be considered while implementing these types of strategies in other settings.

 Cultural values in Mayan culture are different than most Western societies, thus we needed to adapt to this challenge, and the outcome was positive.

# Peer review: Referee response for version 1

**DOI:** 10.5256/f1000research.9111.r13527

**Published:** 2016-08-30

**Authors:** Anna M Stewart Ibarra

**Affiliations:** 1 Center for Global Health and Translational Science and Department of Medicine, State University of New York Upstate Medical University, Syracuse, NY, USA

**Overall: **Good example of intersectoral engagement and innovation in vector surveillance. The ovillanta design, which the authors show to be a highly sensitive vector monitoring tool, is most important element of this publication.  The paper needs to be strengthened through greater review of the literature in the introduction and discussion, clarifying research methods especially social science methods, and clarifying the data shown in the figures. The title is misleading (and some of the text), because the authors do not demonstrate a reduction in *Ae. aegypti* abundance.

**Introduction**

 Paragraph 3. “The rapid spread of *Ae. aegypti *mosquitoes… began around 2000.” I’m not sure that this is correct. I believe that Aedes reinvaded the Americas in the 1970s-80s, and dengue reemerged and spread through the region in the 90s and 2000s. Please check other references.

 Paragraph 4. Strengthen the discussion of Aedes surveillance tools. I suggest discussing currently available ae aegypti surveillance techniques, the role of surveillance in disease control, and limitations of current techniques. Are you comparing stationary vector surveillance/monitoring tools, vector control tools, or integrated surveillance and vector control tools? Traditional ovitraps, human landing catches, and bg sentinel traps all have slightly different objectives. Be sure to clarify this. I would suggest refocusing on the role of ovitraps, and conducting a more comprehensive literature review of different ovitraps currently on the market, their limitations and strengths. 

 I suggest incorporating the paragraph about filtration and recycling into the subsequent paragraph so it is easier for the reader to understand that the current study is based on prior well-developed studies conducted in Canada. This will help with the flow of the intro.

 How did you determine the most attracting solution? Lab based experiments? Please explain or cite prior studies.

 In the introduction, please clarify the objectives of the study, the study design, and the study endpoints (e.g., increased education of health workers, number of Ae. aegypti eggs in XX communities).

**Methods**

 I was a bit confused by the study design. Consider creating a diagram or map that depicts the study design. Why were standard ovitraps used in the intervention community?

 Egg counts are an indirect measure of adult female mosquito abundance (true entomological risk). Egg counts are affected by the number of alternative breeding sites in the environment. Did you account for other factors that could affect egg counts during the study period, such as differences in household characteristics between the treatment and control sites, elimination of containers or other vector control interventions?

 Overall the social science research methods and results lack sufficient detail determine the validity of the results. Three interviews is a small sample size. How do you know these are representative? Given the small number of informants, this information could also be included as part of the local context in the discussion section instead of presented as a result. How did you analyze the qualitative data from focus groups and interviews? What social science research methods were used to analyze the transcribed texts? Were the texts coded? Who did the coding? What software was used? Please include appropriate description of the methods and references.

 Please provide more detail regarding the entomological surveys. Did you empty and collect and count all larvae/pupae from all containers? How were cisterns managed? Did you conduct the entomological surveys in the control neighborhoods to ensure that there were no differences between treatment and control sites? What dates/season did you conduct the surveys? The container positivity will vary greatly by season.

**Results**

 Can you summarize the results of the training evaluation survey in a table?

 Strongly recommend showing summary statistics (maybe from the national census if not available from study households?) to compare the characteristics of the the treatment and control communities or households, to show that there were no major differences in housing conditions, demographics, access to piped water, etc. which could influence differences in the egg count data. In my experience, subtle differences in access to piped water can create completely different vector population dynamics even within the same city.

 Fig 2, left panel. This figure is supposed to show monthly egg counts by site (treatment and control), but the legend indicates trap types rather than site types. This is confusing since both standard ovitraps and ovillantas were used in the intervention site.

 Fig 2, right panel. This figure is supposed to show monthly egg counts by site and by type of trap, but there are 2 lines on the figure rather than 4. Please clarify.

 Fig 3. Why did you fit a linear curve? There appears to be seasonality in the data, indicating a nonlinear seasonal dynamic.

 Please produce publication quality graphics, with consistent formatting and axes (e.g., epi weeks versus months)

 The investigators report that more eggs were collected from the intervention site with ovillantas.  This clearly shows that ovillantas are a more sensitive surveillance tool. I think it is difficult to say whether they are more effective at reducing the mosquito population, unless you have other entomological indicators to show that the mosquito population was suppressed.

**Discussion**

 The investigators indicate that the level of knowledge of community members about viral infections is low. Please show these results in the results section.

 Under study limitations, the major limitation (if the authors are claiming that the ovillanta is an effective vector control tool) is the lack of data on adult *Aedes aegypti* abundance or other entomological indicators.

 The investigators should broaden their discussion to discuss their findings in light of other studies published regarding community dengue perception, alternative ovitrap designs that have been published recently by R. Barrera and others, and the effectiveness of community participation in vector surveillance. The discussion and introduction could be strengthened by a deeper review of the literature.

**Other comments:**

 Please include a map of your study site.

 The authors mention that they limited by lack of climate data. This information is available online. They could download the data and and plot temperature and rainfall with Figure 3, to explain the seasonality.

I have read this submission. I believe that I have an appropriate level of expertise to confirm that it is of an acceptable scientific standard, however I have significant reservations, as outlined above.

# Response

**Published:** 2016-11-30

**Authors:** Gerard Ulibarri

**Affiliations:** Laurentian University, Canada

Title: The title has changed to express these are preliminary results on the approach.

**Introduction**:

**Par. 3**; These statement has been modified and a proper reference added

**Par 4**;The intention of the paper was to showcase the positive preliminary result of the project.  The comparative study of ovitraps in the market should be a full paper on itself, and a new publication is being prepared o that topic.  The authors consider this not to be strictly necessary for this paper, because we are only trying to show that ovillantas are slightly better than standard ovitraps when used in clusters of oviposition traps.

**Attractant solution**: We used a commercial formulation, out of Canada, recommended to attract *Aedes* mosquitoes.  Thus, we cannot describe how the solution was developed in this paper, we only give reference to where we obtained it from.

**Method**;Oviposition count on a standard ovitraps is the standard way of measuring *Aedes* mosquito presence in a neighbourhood.  Thus, having standard ovitraps in the site where the ovillantas were present, seemed the right way to determine the amount of female mosquitoes present in the area.

 But of course, we had the ovillantas as well….which could have served to determine the same, but not recognized as ‘the standard method’

**Egg count**;Traditional cleaning of the neighbourhoods is a standard method employed in Latin America to reduce mosquito breeding sites.  Since this was already implemented in Sayaxche, we only verified that this was a regular exercise.  An it was through out the study.

**Social Scienc**e;We agree, these are only preliminary results, more in depth studies are planned and will be published accordingly.

 Recommendation;We considered that all the neighbourhoods used for the study around the city of Sayaxche have the same (or statistically similar) conditions of piped water, rain water containers, garbage clean up interventions, Health Unit educational information about mosquito issues and interventions.

 National census or data from the local Government is difficult to gather, we are still waiting for data form official sources.

# Peer review: Referee response for version 1

**DOI:** 10.5256/f1000research.9111.r15613

**Published:** 2016-08-25

**Authors:** Leon Eklund Hugo

**Affiliations:** 1 Mosquito Control Laboratory, QIMR Berghofer Medical Research Institute, Brisbane, Qld, Australia

This investigation incorporated three components to improve the control of *Aedes aegypti*, mosquito vector of dengue, Chikungunya and Zika viruses, in a remote urban community in Guatemala. The three components were; use of web-based training in vector control for local health personnel, the development of mosquito ovitraps made locally out of used tires and evaluation in a trapping survey and community engagement activities. The authors are commended on the performance of a mosquito trap made from a common waste item that normally contributes to mosquito production. The new traps, referred to as ovillantas, caught more eggs per site, per device, than standard ovitraps.  

 Although the title refers to the control of *Ae. aegypti*, which is the theme throughout the manuscript, the studies presented are not specifically tailored to measuring the success of the components in the context of *Ae. aegypti *control (mosquito population reduction). For component one; web-based education of health workers; a knowledge, action and practice (KAP) survey of the knowledge of health workers before they undertook training could have assisted to determine the changes due to the training module. For component two; data for the counts of mosquito larvae/pupae in different mosquito traps over time is presented within treatment communities. While this provides promising data on the effectiveness of ovillantas as trapping tools, there are no indication that the trapping had the effect of reducing the mosquito population. Ecological ovillantas may prove to be excellent tools for monitoring mosquito population numbers in resource limited settings, however more work is needed to establish their ability to reduce mosquito population sizes.  The authors attempted to asses the effect of their intervention on mosquito control by examining Dengue seroprevalence data, however low case numbers were reported in official records and these are likely to be underestimates, limiting this approach. Adult mosquito trapping would be another avenue to monitoring mosquito population changes. For component three, several themes were identified among socioeconomic issues affecting mosquito control, however future studies will be required to measure resulting improvements in community engagement.

 In summary, a multi-component strategy towards the control of *Ae. aegypti* is presented that incorporates training of health workers in vector control, a new mosquito ovitrap constructed from recycled materials and community engagement activities. While an increase in trapping efficacy is demonstrated for the ovillantas, it is difficult to gauge the effectiveness of these components on *Ae. aegypti *control from this study alone. The title and theme may be better presented as "development of strategies *towards* the control of *Ae. aegypti*". I am hopeful that improved *Ae. aegypti *control can be demonstrated from future application of these interventions.

I have read this submission. I believe that I have an appropriate level of expertise to confirm that it is of an acceptable scientific standard, however I have significant reservations, as outlined above.

# Response

**Published:** 2016-11-30

**Authors:** Gerard Ulibarri

**Affiliations:** Laurentian University, Canada

Although we tend to agree with the Mr. Hugo (reviewer),we clarify that this is a report on preliminary data.  Entomological studies are necessary in order to establish with certainty that the viral infection has been reduced.  Adult traps do not work very well in *Aedes* spp. mosquitoes (reason for the use of standard ovitraps to monitor the presence of *Aedes* females in the study area). Nevertheless adult traps are planned for the near future studies, something very difficult to achieve in remote communities where commercial carbon dioxide is not easy to access.  A new trap is being designed to overcome this difficulties, and results will be reported in due time.

**In summary; **Title has been modified to express that these are  ‘preliminary results”, more detailed data will be provided in due course

# Peer review: Referee response for version 1

**DOI:** 10.5256/f1000research.9111.r14070

**Published:** 2016-05-31

**Authors:** Laith Yakob

**Affiliations:** 1 Department of Disease Control, London School of Hygiene & Tropical Medicine, London, UK

**Title and Abstract: **I think these are both a little misleading. The integrated intervention was not evaluated in such a way that would allow for rigorous evaluation (there were too few trial arms) – I think the presented results can only be used to compare the two different trap types. The sentence in the abstract that indicates the higher mosquito egg collections found using the novel trap is also misleading – analysing total egg counts over the course of the experiment actually indicated higher captures with the standard ovitrap.

**Article content:** There are major issues with the study design. Not least of all, it does not allow for the assessment of any additive benefits from community engagement and healthcare worker education. The authors tested their new ‘ovillantas’ trap against standard ovitraps. Unfortunately, they only used lure-baited water in their novel trap meaning any difference found between trap types could just as easily be a result of the lure as it was the new trap design. Although cost was mentioned several times in the study, no indication was provided of the relative costs of the new tested trap (plus lure and construction time) versus the standard trap and so no conclusions could be drawn pertaining to cost effectiveness.

 Recycling the water in the traps is a good idea in the short term because natural chemicals emitted by ovipositing mosquitoes are attractive to future ovipositors. However, there must be an optimum duration over which water can be recycled, beyond which the build-up of algae etc would actually be unattractive to ovipositing females. This was never mentioned.

 Lack of internet access in this remote population is something that perhaps should have been identified as a major study limitation before the attempted use of web-based education services.

 After community participation inevitably wanes and the eggs are no longer collected and routinely removed/destroyed, the ovillantas will provide ideal breeding grounds for disease vectors. Part of the study involved community education on eliminating breeding grounds from participant houses but these ‘traps’ sound as though they themselves will become ideal larval sites in time. The only ways around this would be the removal of traps by trained personnel over a predetermined time period or the continual surveillance of the local communities’ upkeep of the traps – both expensive and unlikely to occur.

 Count data were analysed using t-tests. Count data are typically not normally distributed (typically they are Poisson or negative/binomial distributed) and would require alternative analysis methods.

 Minor issues in the content include the assertion that ovitraps have previously been shown to significantly reduce adult mosquito numbers – to the best of my knowledge this is not the case. Also, the authors mention that the 500m dispersal ability of *A. aegypti *informed the distribution of the intervention households (no more than 50 metres apart) but I neither understand this logic nor agree with this very-much-upper-estimate in *A. aegypti *mosquito movement.

**Conclusions:** Overall, I’m left with questions over the usefulness of this new tool relative to the standard trap – depending on how the authors analysed their data they showed improved captures with both traps (and neither analysis was conducted rigorously). I also am left unsure of the additional benefits in terms of community participation or education/training because the study design did not allow for these to be tested independently.

**Data: **The data appear to be presented clearly and are in a format that allows for their future use by other scientists.

I have read this submission. I believe that I have an appropriate level of expertise to confirm that it is of an acceptable scientific standard, however I have significant reservations, as outlined above.

# Response

**Published:** 2016-11-30

**Authors:** Gerard Ulibarri

**Affiliations:** Laurentian University, Canada

**Title and Abstract**;The title has been modified to clarify that these are preliminary results shared with the public due to the Zika presence.  More trials are planned when funding is granted.  In the mean time, this is an approach which is working and should be used to help people protect themselves.

 One of the novelties here was that people from the community did participate actively, using a novel approach made out of items from their backyard.

 Higher mosquito egg count was achieved on site with ovillantas against site with only standard ovitrap.  Although, the reviewer is right that the ovitrap in ovillanta site collected more than the ovitrap alone.  We are investigating this phenomenon and believe is due to the ‘skip oviposition’ nature of the *Aedes* mosquito.

**Article content**; We also compared water with ovillantas, against standard ovitraps, and at all time the ovillantas collected about 2.5 times more eggs than standard ovillanta (data not shown).

 ​​​​​​We did find algae problem but in very few ovillantas.  The fact of filtering and recycling the solution seemed to keep the solution clean for extended period of time.  When an ovillanta was polluted, then the whole solution was replaced with fresh water plus attractant solution.

 ​​​​​​Internet accessibility at the Health Center was identified prior to implementation of the study.  What was a problem, and we did not see it at the beginning, was that not everybody has access to internet in their homes, and internet is extremely slow in the region.  Thus, it was necessary that every single person travel to the Health Center for the ‘lessons’, an inconvenience to those far from the Center.

**Community participation**; Absolutely, the same would happen if standard ovitraps were used.  For this reason, the Health personnel was trained to supervise the surveillance and cleanliness of the ovillantas in a regular basis while gathering the information on egg count (pellon paper exchange).  If the participant failed to maintain the ovillanta, this was removed from their premises or the Health personnel took over the maintenance.  Very few instances of this happened.

 t-tests; Other methods will be used and compared when more data is available.  For the time being, we considered that the t-tests were enough to show the significant difference provided by the use of ovillantas against standard ovitraps.

**Conclusions**: This has been a very difficult, three-prong, study, and the paper reflects the first data produced, more will be produced in due time.  The Health personnel showed improvement after their Internet-based course, the community accepted the ovillantas very well and participated (something new), and the cluster of ovillantas collected more eggs than the isolated standard ovitraps. Those are the facts. 

 We are still studying how each one of the interventions influenced the outcome of the whole intervention.  In this report, we have presented the results to the holistic approach.  More questions will be answered and data will be presented in due time
